# Ultrasmall CsPbBr_3_ Blue Emissive Perovskite Quantum Dots Using K-Alloyed Cs_4_PbBr_6_ Nanocrystals as Precursors

**DOI:** 10.1021/acsenergylett.4c00693

**Published:** 2024-04-23

**Authors:** Clara Otero-Martínez, Matteo L. Zaffalon, Yurii P. Ivanov, Nikolaos Livakas, Luca Goldoni, Giorgio Divitini, Sankalpa Bora, Gabriele Saleh, Francesco Meinardi, Andrea Fratelli, Sudip Chakraborty, Lakshminarayana Polavarapu, Sergio Brovelli, Liberato Manna

**Affiliations:** † CINBIO, Department of Physical Chemistry, Materials Chemistry and Physics Group, Universidade de Vigo, Campus Universitario As Lagoas-Marcosende, 36310 Vigo, Spain; ‡ Nanochemistry, Istituto Italiano di Tecnología, Via Morego 30, 16163 Genova, Italy; § Dipartimento di Scienza dei Materiali, Università degli Studi di Milano-Bicocca, Via R. Cozzi 55, 20125 Milano, Italy; ∥ Electron Microscopy and Nanoscopy, Istituto Italiano di Tecnología, Via Morego 30, 16163 Genova, Italy; ⊥ Dipartimento di Chimica e Chimica Industriale, Università di Genova, 16146 Genova, Italy; # Material Characterization Facility, Istituto Italiano di Tecnologia Via Morego 30, 16163 Genova, Italy; ∇ Materials Theory for Energy Scavenging (MATES) Lab, Department of Physics, Harish-Chandra Research Institute (HRI), A C.I. of Homi Bhabha National Institute (HBNI), Jhunsi, Prayagraj 211019, India

## Abstract

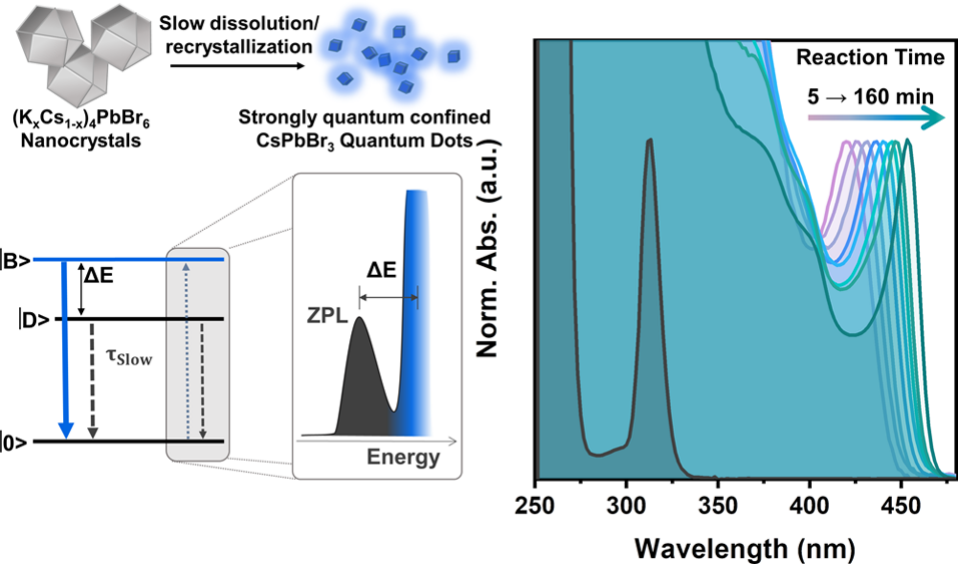

We report a colloidal synthesis of blue emissive, stable cube-shaped CsPbBr_3_ quantum dots (QDs) in the strong quantum confinement regime via dissolution–recrystallization starting from pre-syntesized (K_*x*_Cs_1–*x*_)_4_PbBr_6_ nanocrystals which are then reacted with PbBr_2_. This is markedly different from the known case of Cs_4_PbBr_6_ nanocrystals that react within seconds with PbBr_2_ and get transformed into much larger, green emitting CsPbBr_3_ nanocrystals. Here, instead, the conversion of (K_*x*_Cs_1–*x*_)_4_PbBr_6_ nanocrystals to CsPbBr_3_ QDs occurs in a time span of hours, and tuning of the QD size is achieved by adjusting the concentration of the precursors. The QDs exhibit excitonic features in optical absorption that are tunable in the 420–452 nm range, accompanied by blue photoluminescence with quantum yield around 60%. Detailed spectroscopic investigations in both the single and multiexciton regime reveal the exciton fine structure and the effect of Auger recombination of these CsPbBr_3_ QDs, confirming theoretical predictions for this system.

Colloidal lead halide perovskite nanocrystals, with CsPbBr_3_ being the flagship stable material of the class, are a family of semiconductor materials with great potential for optoelectronic applications, such as light-emitting diodes (LEDs), quantum light emission, scintillators, and lasers, thanks to their excellent optical properties, which include narrow emission in the visible range of the spectrum with near unity photoluminescence quantum yield (PLQY).^1−3^ In CsPbBr_3_, the crystal structure is based on a 3D network of corner-sharing [PbBr_6_]^4–^ octahedra that are charge-balanced by Cs^+^ cations positioned in the voids created by such a network. Other Cs–Pb–Br phases are known, such as non-luminescent Cs_4_PbBr_6_ and CsPb_2_Br_5_, the former being made of disconnected [PbBr_6_]^4–^ octahedra (hence a “0D” phase) separated by Cs^+^ cations and the latter made of layers of connected [Pb_2_Br_5_]^−^ anions alternating with layers of Cs^+^ cations (hence a “2D” phase).^4−9^ It has been shown that the three phases can be interconverted, for example, by PbBr_2_ addition (0D → 3D; 3D → 2D)^6,10,11^ or by CsBr extraction (0D → 3D; 2D → 3D).^12,13^ In this work, we show that in colloidal nanocrystals the 0D → 3D interconversion can be remarkably slowed down, such that starting from 0D Cs_4_PbBr_6_ nanocrystals, these are slowly used up to synthesize ultrasmall CsPbBr_3_ nanocrystals in the strong quantum confinement regime.

For colloidal semiconductor nanocrystals to be classified as “quantum dots” (QDs) their size, *d*, should be comparable to or smaller than the exciton Bohr diameter (*d*_B_) of that material, which is around 7 nm for CsPbBr_3_.^14^ Conventional methods for the colloidal synthesis of CsPbBr_3_ nanocrystals usually deliver relatively “large” CsPbBr_3_ nanocrystals (with *d* ∼ 8 nm or beyond)^14,15^ in a time range of seconds.^2^ It is possible to grow smaller nanocrystals, for example, by reducing the reaction temperature,^16−20^ modulating the precursor ratios,^18^ working in an environment with a high concentration of ligands,^19^ or using “bulky” ligands with long alkyl chains,^20,21^ but only a few groups have succeeded in preparing stable CsPbBr_3_ QDs with sizes below 5 nm.^18−20,22−27^ Dong et al. demonstrated the synthesis of CsPbBr_3_ QDs below 5 nm size by tuning the Pb/Br ratio using ZnBr_2_ as an extra source of Br.^18^ More recently, Akkerman et al. were able to exert tighter control on the nanocrystal growth kinetics by using tryoctylphosphine oxide (TOPO) and diisooctylphosphinic acid (DOPA) as weak coordinating ligands, achieving a much slower growth rate compared to synthesis schemes that use more conventional ligands such as oleylammonium (OLAm) and oleate (OL). They also managed to isolate CsPbBr_3_ QDs smaller than 4 nm by stabilizing them with lecithin, a long chain zwitterionic ligand.^28^ A similar approach was employed by Bi et al. to stabilize and isolate CsPbBr_3_ QDs through a postsynthetic ligand exchange with phenylethylamine.^26^ There have also been reports on the synthesis of ultrasmall CsPbBr_3_ nanocrystals with intermediate size between molecules and QDs, usually named “nanoclusters”.^29−32^ However, over time, they tend to aggregate. These clusters have mainly been used as “precursors” to prepare CsPbBr_3_ nanocrystals of larger sizes or different geometries^31,33−35^ or even to grow perovskite-based heterostructures.^34,36−40^

Another way of slowing down the nucleation and growth of CsPbBr_3_ nanocrystals was identified by Su et al.^41^ through the addition of Ag^+^ ions. Monovalent cations smaller than Cs^+^ (such as Ag^+^ or alkali metals) cannot penetrate into the crystal lattice in significant amounts to form alloy compositions, and at best they can enter in small amounts as dopants.^42,43^ On the other hand, alkali metal cations such as K^+^ and Na^+^ can bind to the surface of perovskite nanocrystals (most likely by forming stable metal–halide bonds).^44−46^ The presence of surface-bound alkali metal cations may suppress the formation of trap states and consequently the ion migration but can also prevent the aggregation/coalescence of nanocrystals. For example, platelet-shaped CsPbBr_3_ nanocrystals were better able to preserve their shape and optical characteristics under photo and thermal stress over time when treated with K^+^ cations, compared to untreated platelets.^45^

Here, we capitalize on the various previous findings, as highlighted above, and report a new route to synthesize stable blue emissive CsPbBr_3_ QDs in the strong quantum confinement regime, starting from 0D (K_*x*_Cs_1–*x*_)_4_PbBr_6_ nanocrystals that were used as reactants. A first key discovery of our work is that Cs_4_PbBr_6_ nanocrystals can be alloyed with a substantial amount of K^+^ ions, reaching close to 20% replacement of Cs^+^ with K^+^ ions (Figure 1). This is markedly different from the case of CsPbBr_3_ discussed above, in which alloying with K^+^ is not observed. Crucial to this work, these (K_*x*_Cs_1–*x*_)_4_PbBr_6_ nanocrystals are much less reactive than the unalloyed Cs_4_PbBr_6_ ones toward PbBr_2_. The addition of PbBr_2_ to Cs_4_PbBr_6_ nanocrystals is known to rapidly transform them to CsPbBr_3_ nanocrystals,^6^ whereas the (K_*x*_Cs_1–*x*_)_4_PbBr_6_ nanocrystals are much less reactive and follow a dissolution–recrystallization reaction pathway: they essentially act as reservoirs, steadily getting dismantled and releasing monomers for the slow nucleation and growth of CsPbBr_3_ QDs in a size range between *d* = 3 nm and *d* = 3.5 nm. These QDs, once isolated and purified, remain stable and do not evolve further in size nor do they aggregate. This is markedly different from previous works on the synthesis of QDs which required the addition of bulky ligands (such as lecithin^28^ or phenylethylamine^26^) to preserve colloidal stability once the QDs were isolated from the reaction environment. At present, the reasons for the increased stability of our QDs are unclear, and we can only speculate that it might stem from the presence of trace amounts of K^+^ ions on the QD surface, although this remains a hypothesis, as the concentration of K^+^ ions in the final sample is below the experimental error.

**Figure 1:**
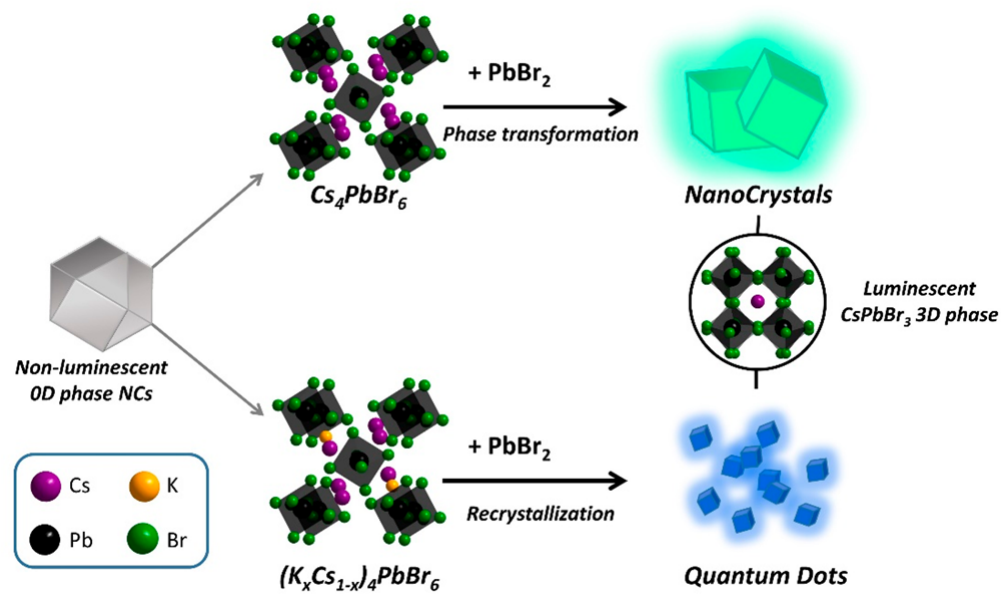
Sketch of the conversion of 0D-phase Cs_4_PbBr_6_ nanocrystals to 3D-phase CsPbBr_3_ nanocrystals/QDs by PbBr_2_ addition. Top: unalloyed Cs_4_PbBr_6_ nanocrystals yield large CsPbBr_3_ nanocrystals through a fast reaction with PbBr_2_, while K-alloyed (K_*x*_Cs_1–*x*_)_4_PbBr_6_ nanocrystals yield <3.5 nm size CsPbBr_3_ QDs through a slow dissolution–recrystallization process.

The much higher stability of these QDs compared with previously reported materials in the same *d*-range enabled a more careful study of the photophysics of CsPbBr_3_ in the strong quantum confinement regime. We therefore investigated both the single and multiexcitonic behavior for QDs with *d* ∼ 3.5 nm. Cryogenic time-resolved photoluminescence (PL) experiments, corroborated for the first time for halide perovskite QDs by fluorescence line-narrowing (FLN) measurements in both continuous wave (*cw*) and time-resolved modes, confirm the characteristic signatures of a dark exciton state separated from the overlying bright exciton state by a dark–bright splitting energy Δ_DB_ = 15 meV for *d* = 3.5 nm, in full agreement with the theoretical predictions of Efros and co-workers,^47^ and extending the size range for which the exciton structure is now known. Also consistently, control samples of the tiniest QDs we could synthesize by conventional hot injection methods (*d* ∼ 4 nm) featured a smaller Δ_DB_ (10.4 meV), and growing larger particles further modified the exciton fine structure, leading to a level crossover driven by the Rashba effect, resulting in a lower lying bright exciton state and accelerated radiative decay at low temperatures. We also performed transient absorption (TA) measurements to assess the influence of non-radiative Auger recombination on the biexciton yield in strongly confined QDs. The biexciton lifetime for *d* = 3.5 nm QDs is estimated in 8.2 ps, which agrees well with the projected volume-scaling and sets a new small-size limit to our estimation of the Auger process in CsPbBr_3_ QDs.

The starting Cs_4_PbBr_6_ nanocrystals were synthesized following a previously reported hot injection method (see the experimental section in the Supporting Information for more details).^6^ The crude nanocrystal solution was purified by centrifugation (without using any antisolvent), and the precipitated nanocrystals were then redispersed in toluene. The K-alloyed (K_*x*_Cs_1–*x*_)_4_PbBr_6_ nanocrystals were obtained from this sample by a partial cation exchange reaction (Figure 2a) which was performed by adding a solution of potassium oleate to the colloidal Cs_4_PbBr_6_ nanocrystal dispersion and letting the mixture react for a few seconds. This was followed by the addition of ethyl acetate (EtOAc) as antisolvent, precipitation of the nanocrystals via centrifugation, and their redispersion in a solution of oleylamine and oleic acid in toluene to compensate for the partial loss of ligands that is likely to occur during the purification. The procedure was repeated three times to guarantee the maximum incorporation of potassium. The UV–vis absorption spectra of the nanocrystals before and after the reaction with K-oleate are shown in Figure 2b. After the reaction, the characteristic excitonic peak at ∼314 nm corresponding to the Cs_4_PbBr_6_ nanocrystals was shifted to slightly higher energies (∼311 nm). Neither of the samples emits a light. Based on transmission electron microscopy (TEM) and high annular dark field (HAADF)-scanning TEM, the hexagonal shape (in projection) of the starting Cs_4_PbBr_6_ nanocrystals was preserved after the partial cation exchange reaction (Figure 2c–f, Figures S1 and S2), and their average size slightly decreased from 17.8 to 17.0 nm (see also Figures S1 and S2), caused by a lattice contraction by the introduction of potassium, due to the smaller ionic radius of K^+^ with respect to Cs^+^ (1.38 vs 1.68 pm),^48^ and also some etching of the nanocrystals, due to their exposure to a mild polarity during the several cycles of washing with EtOAc.

**Figure 2:**
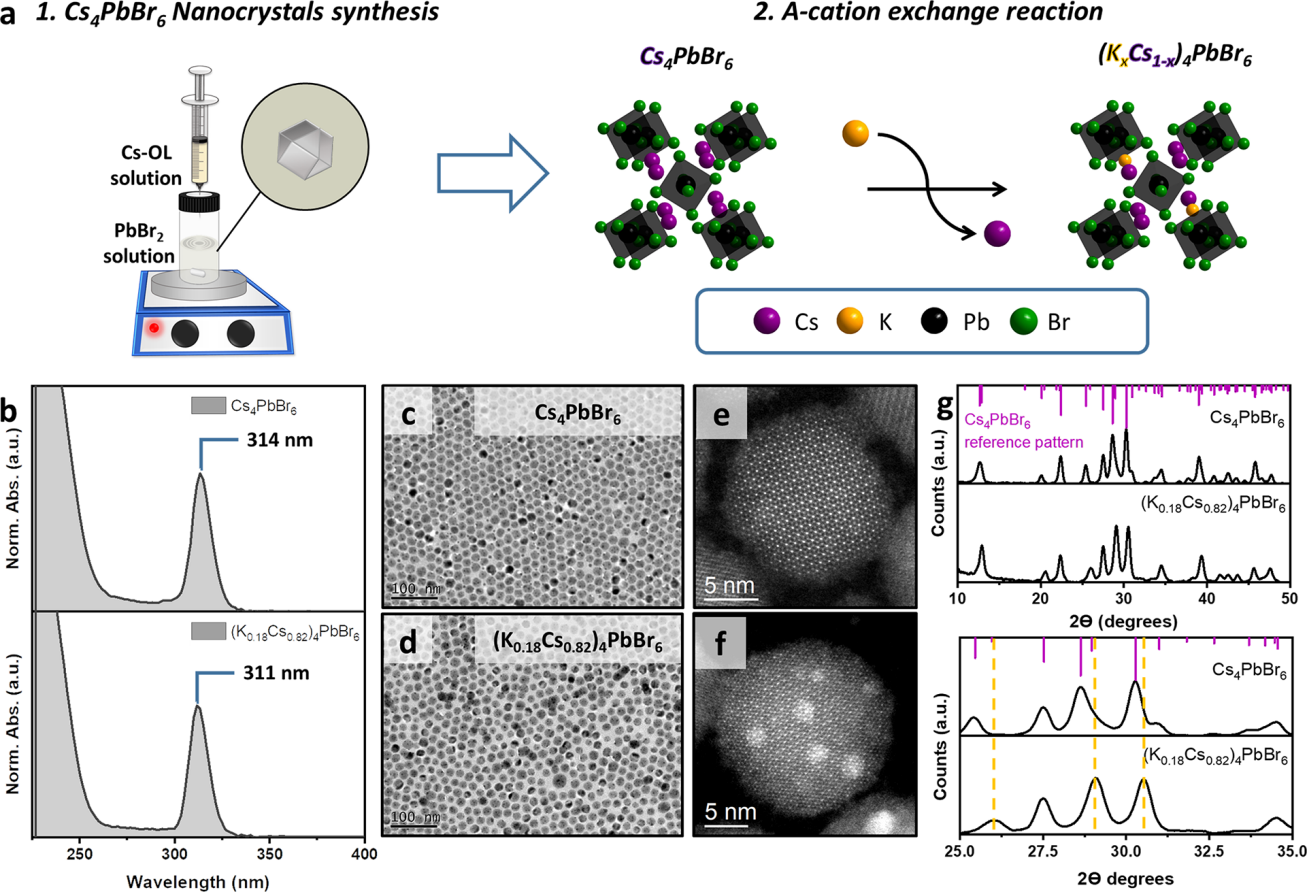
(a) Schematic illustration of the synthesis of Cs_4_PbBr_6_ nanocrystals by the hot injection of a Cs-oleate (Cs-OL) solution into a vial containing the PbBr_2_ solution. (b–g) UV–vis absorption spectra (b), low magnification TEM images (c, d), corresponding high resolution HAADF-STEM images from the [001] zone axis (e, f), and XRD diffraction patterns (g) of Cs_4_PbBr_6_ nanocrystals and the corresponding (K_*x*_Cs_1–*x*_)_4_PbBr_6_ nanocrystals obtained by partial Cs^+^ → K^+^ cation exchange. The scale bar in panels c and d is 100 nm. At the bottom of panel (g), the XRD pattern in the region between 25° and 35° highlights the peak shifts in the alloyed sample compared to the initial sample.

The X-ray diffraction (XRD) pattern of the pristine sample (Figure 2g) matched with the typical features of the trigonal R3̅c space group and was in agreement with previous reports.^49^ After the reaction with K^+^, the XRD peaks were shifted to higher angles (see the zoomed diffraction pattern in the bottom panel of Figure 2g). This is ascribed to the contraction of the cell due to the partial replacement of Cs^+^ with K^+^ (Figure 2a and g). The calculated lattice parameters of the pristine and final alloy samples (Figure S3) confirm a 1.1% decrease in the unit cell volume following the exchange reaction. The elemental composition of the two samples was determined by scanning electron microscopy-energy dispersive X-ray spectroscopy (SEM-EDS) and HAADF-STEM-EDS (see Figures S4–S7). According to the analyses, the pristine sample matches a Cs_4_PbBr_6_ stoichiometry and the final alloy sample matches a (K_0.18_Cs_0.82_)_4_PbBr_6_ one. Hence the Cs^+^ → K^+^ exchange was not complete and most likely reached a thermodynamic equilibrium. The difficulty in achieving a more extensive exchange is also evident from the fact that there is no known stable K_4_PbBr_6_ phase.

We performed density functional theory (DFT) calculations to elucidate the effect of K^+^ alloying on the electronic structure of Cs_4_PbBr_6_ (Figure S8). We systematically explored all of the possible K^+^ substitution positions in order to find the lowest-energy configuration. Due to the resulting combinatorial complexity, the simulated doping concentration was limited to 12.5%, which is however sufficient to study the effect of K^+^ alloying. Upon K^+^ alloying, the band gap increases from 3.91 to 3.96 eV, in good agreement with the experimental findings (Figure 2b). The inspection of the density of states reveals that neither Cs^+^ nor K^+^ participates in the electronic states near the band edges (Figure S9). The slight band gap widening upon K^+^ doping can thus be ascribed to the resulting lattice contraction (−2.2% in volume according to the DFT). Both the valence and conduction bands display a dominant contribution from Pb orbitals and a smaller but significant contribution from Br orbitals. The comparison with 3D lead halide perovskites suggests that the valence and conduction bands are formed by Br(p)–Pb(s) and Br(p)–Pb(p) antibonding states, respectively.^50,51^ However, unlike 3D perovskites, in 0D perovskites the corresponding bands are quite flat (low dispersion) since they localize in isolated [PbBr_6_]^4−^ octahedra (Figure S10). This also indicates much higher electron and hole effective masses in 0D perovskites. Finally, we note that in both doped and undoped Cs_4_PbBr_6_ the band gap is indirect (Figure S10), possibly explaining the absence of photoluminescence in these compounds.

We carried out analyses via nuclear magnetic resonance (NMR) spectroscopy to characterize the ligand shell of the two systems (the non-doped Cs_4_PbBr_6_ nanocrystals and the (K_*x*_Cs_1–*x*_)_4_PbBr_6_ ones). Details are reported in the Supporting Information (SI) (Figures S11–S14). These analyses evidenced a marked difference in the composition and density (SEq. 1 and 2) of the ligand shell for the two systems. The Cs_4_PbBr_6_ nanocrystals were characterized by a densely packed ligand shell (∼3.5 ligands/nm^2^) constituted by 78% oleate and 22% oleyl ammonium. On the other hand, the (K_*x*_Cs_1–*x*_)_4_PbBr_6_ nanocrystals were characterized by a significantly less packed ligand shell (∼2.3 ligands/nm^2^) again mainly made of oleate (81%), with only 19% oleylammonium. These marked differences in the ligand shell density for the two systems are probably responsible for their much different reactivities toward PbBr_2_, as discussed in the following section.

The reactivity of the synthesized Cs_4_PbBr_6_ and (K_0.18_Cs_0.82_)_4_PbBr_6_ nanocrystals in toluene toward a PbBr_2_ solution was investigated regarding their chemical and morphological transformations. The Cs_4_PbBr_6_ nanocrystals were already reactive at room temperature and quickly converted to 12.2 nm cube-shaped CsPbBr_3_ nanocrystals having a strong green emission (Figure S15), as already shown by our group in a previous work.^6^ The (K_0.18_Cs_0.82_)_4_PbBr_6_ nanocrystals were instead much less reactive. Mixing the (K_0.18_Cs_0.82_)_4_PbBr_6_ nanocrystal solution with the PbBr_2_ solution at room temperature only produced an absorption spectrum that was the sum of the two components (Figure 3a, second panel from the top, and Figure 3b).

**Figure 3:**
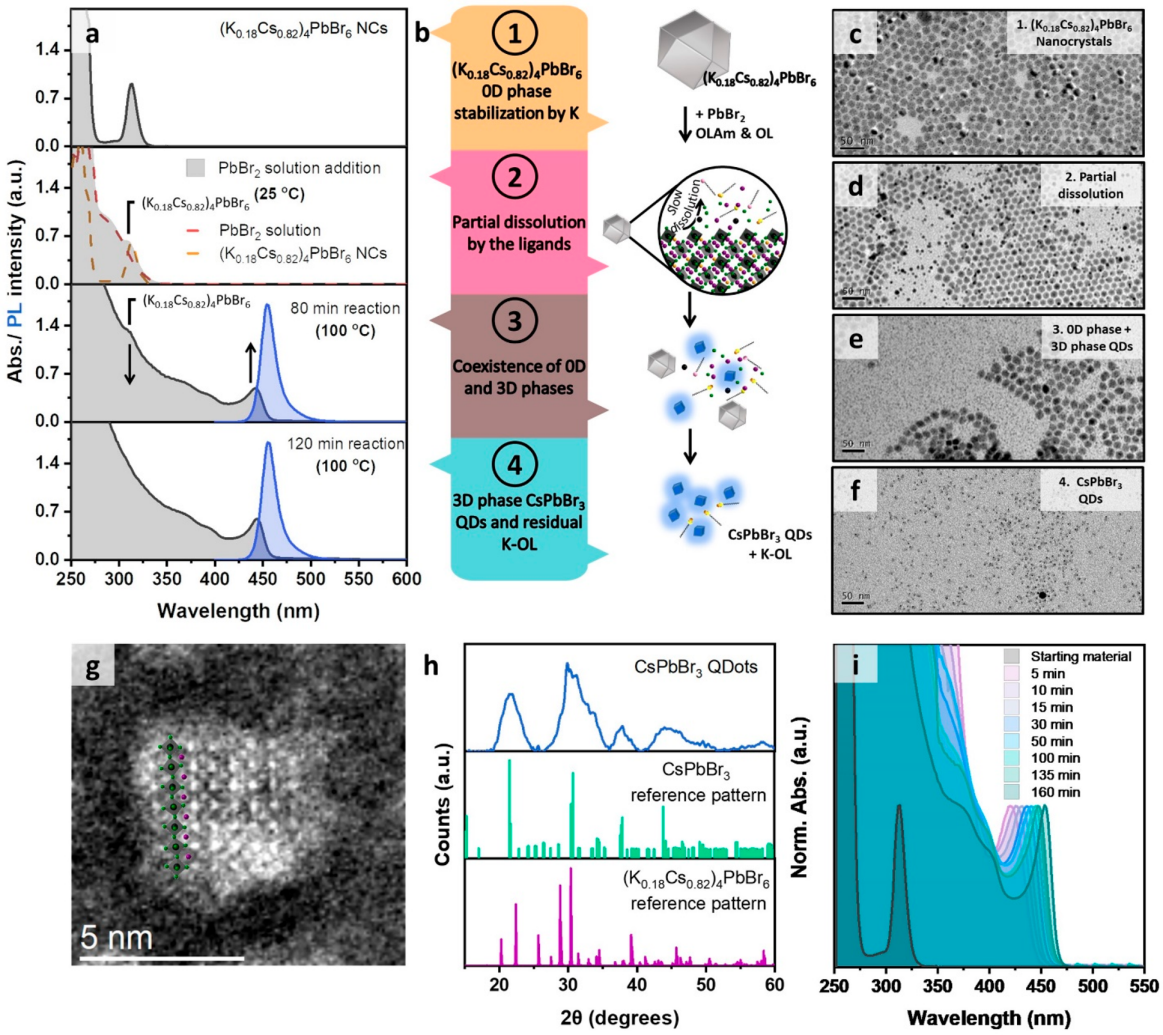
(a) UV–vis absorption and PL spectra of the 0D phase (K_*x*_Cs_1–*x*_)_4_PbBr_6_ conversion to 3D phase CsPbBr_3_ QDs at different reaction stages. (b) Schematic illustration of the proposed reaction mechanism. (c–f) TEM images of the 0D phase (K_*x*_Cs_1–*x*_)_4_PbBr_6_ conversion to 3D phase CsPbBr_3_ QDs at different reaction stages. (g) High resolution HAADF-STEM image of a representative QD sample. The images were acquired by using a low electron dose to avoid the degradation of the particles due to the beam sensitivity of the material. The dimensions of the inorganic core along one edge of the cube correspond to about 6 unit cells (∼3.5 nm), based on the number of atom columns with higher contrast (Pb–Br columns). (h) XRD diffraction pattern of the 3.5 nm CsPbBr_3_ QDs (top panel) and comparison to (K_*x*_Cs_1–*x*_)_4_PbBr_6_ and CsPbBr_3_ reference patterns. (i) UV–vis absorption spectra of CsPbBr_3_ QDs of different sizes obtained at 100 °C using different reaction times.

To observe the evolution in the new optical features, the solution had to be heated to 100 °C, after which the optical absorption features typical of very small CsPbBr_3_ nanocrystals started to emerge. This can be seen in Figure S16a, which is a zoomed-in view of the blue region of the spectra. At the same time, an emission feature in the green region of the spectrum was seen, indicating the concomitant presence of a small fraction of larger nanocrystals, which, however, almost completely disappeared at later reaction times (Figure S16b). It is important to note that, while these features emerged and evolved, we could still monitor the persistence in the optical absorption spectrum of the excitonic band at ∼311 nm assigned to the (K_0.18_Cs_0.82_)_4_PbBr_6_ nanocrystals, which however steadily decreased in intensity. This indicates the coexistence of the two materials: the initial (K_0.18_Cs_0.82_)_4_PbBr_6_ nanocrystals, steadily being consumed, and the newly formed CsPbBr_3_ QDs (Figure 3a, third panel from the top, and Figure 3b). It was not until after 2 h of reaction that the signature of the (K_0.18_Cs_0.82_)_4_PbBr_6_ nanocrystals disappeared from the absorption spectrum (Figure S17 and Figure 3a, fourth panel from the top). At this point, the absorption and PL spectra evidenced the presence of a monodisperse population of CsPbBr_3_ QDs with a sharp excitonic band at ∼445 nm and a narrow blue PL emission peak at 455 nm with a full width at half-maximum of 15 nm (Figure 3a, fourth panel).

This and the different stages of the reaction depicted in Figure 3b were also corroborated by the TEM analysis of the initial, intermediate, and final samples (Figure 3c–f) and the corresponding XRD pattern of the final product (Figure 3h). In XRD, the intermediate samples evidenced the presence of both the 0D and 3D phases (Figure S18), whereas in the final sample only the 3D phase was present (Figure 3h). Importantly, the broadening of the XRD peaks due to the restricted number of reflecting planes corroborates the small size of these QDs. After 120 min, the reaction was completed and quenched in an ice–water bath. The solution had a yellow color and was colloidally stable. Due to the high concentration of ligands, the final sample was easily purified by their precipitation with antisolvents and then redispersed in toluene. The peak positions in the XRD pattern of this final sample are fully in line with the pure CsPbBr_3_ perovskite phase, indicating that the QDs do not incorporate any meaningful amount of K^+^. This was also corroborated by elemental analysis via energy dispersive X-ray spectroscopy in scanning electron microscopy (SEM-EDS), which did not reveal any K^+^ trace. Yet, we cannot exclude a small, residual amount of K^+^ ions on the surface of the QDs. An atomic resolution HAADF-STEM image of a representative QD from this sample is reported in Figure 3g.

The sluggish reactivity of the (K_0.18_Cs_0.82_)_4_PbBr_6_ nanocrystals toward PbBr_2_ compared to the pristine Cs_4_PbBr_6_ nanocrystal case suggests that the incorporation of K^+^ ions in the 0D phase decreases their reactivity. This in turn can be ascribed to a different binding strength of the ligands, a different packing density of ligands, or yet a decreased propensity of PbBr_2_ species to intercalate in the (K_0.18_Cs_0.82_)_4_PbBr_6_ alloy phase due to the overall decrease in lattice parameter. We carried out further studies on the 0D → 3D conversion synthesis for better understanding of the mechanism. The time it takes for the (K_0.18_Cs_0.82_)_4_PbBr_6_ nanocrystals to be completely depleted to nucleate and simultaneously grow into CsPbBr_3_ QDs should likely be dependent on their initial concentration. Thus, in principle, the higher the concentration of these initial nanocrystals (hence their availability in the reaction medium), the longer it will take for them to be consumed, and the larger will be the size of the final CsPbBr_3_ QDs at that stage. To validate this hypothesis, several syntheses were performed using different volumes of (K_0.18_Cs_0.82_)_4_PbBr_6_ nanocrystal precursor, while keeping all the other reaction parameters constant (see Table S1 and experimental section in the SI for more details). We indeed observed that the higher the volumes of (K_0.18_Cs_0.82_)_4_PbBr_6_ nanocrystals, the longer the reaction times required to consume all of the precursor material. The UV–vis absorption spectra of the as-synthesized QDs (Figure 3i) exhibit sharp and tunable excitonic peaks from ∼420 to 452 nm (Figure 3i) for the different reaction times. The samples exhibit emission peaks ranging from ∼444 to ∼462 nm, with a full width at half-maximum (fwhm) between 24 and 15 nm, respectively (Figure S19), and PLQY values in the range of 60–65%. Only for short reaction times could we observe emission features in the green, stemming from contamination of larger CsPbBr_3_ nanocrystals, as mentioned earlier. This feature however disappeared for reaction times longer than 30 min. However, for reaction times longer than 160 min, the high concentration of CsPbBr_3_ QDs in solution, along with the depletion of precursors (all the 0D nanocrystals were consumed at this time), initiated an Ostwald ripening process that eventually led to the formation of large green emissive CsPbBr_3_ nanocrystals. Consistent with the size-independent PLQY values of the QD, the fluorescence dynamics was found to be essentially constant with increasing *d* (Figure S20), with mono/biexponential decays with lifetimes around 4.5 ns. As a note, our method allows a relatively narrow range of tunability in the optical features (420–452 nm, see Figure 3i) that indicates a small tunability in size of the QDs in a size range that peaked around 3–3.5 nm.

The stability of a sample of CsPbBr_3_ QDs was monitored by UV–vis absorption and PL spectroscopy for more than 1 year (Figure S21) in a closed vial under air. After this time, the QDs still exhibited strong blue emission (Figure S21, inset), although in the meanwhile the PL peak had shifted from ∼455 to ∼465 nm. The absorption spectra as well had shifted by a comparable amount, while the absorbance intensity was barely affected.

CsPbBr_3_ QDs with *d* < 4 nm have been less explored than larger CsPbBr_3_ nanocrystals. Currently, exciton fine structure studies for particles in the strong confinement regime are very scarce^24,52−54^ and so are investigations of the multiexcitonic recombination.^55^ To fill this gap, we thus interrogated both the single and multiexciton photophysics by time-resolved PL as a function of temperature also in fluorescence line narrowing (FLN) configuration and in the presence of strong magnetic fields, and by TA experiments in the single vs. multiexciton regime. The PL decay curves of CsPbBr_3_ QDs synthesized with our new approach, with *d* = 3.5 nm (Figure 3g), at decreasing temperature (300–2 K) are depicted in Figure 4a. The corresponding time and spectrally resolved PL decays at room temperature are reported in Figure S22 showing uniform emission decay across the whole PL spectrum. Consistent with previous reports,^47,56−58^ cooling the CsPbBr_3_ QDs from 300 to 2 K progressively slowed down the PL dynamics, turning from an essentially single exponential behavior to a strongly biexponential kinetics dominated by a microsecond slow recombination tail. To better monitor the PL dynamics with temperature, we extracted the PL decay rates (*k*) and reported them in the inset of Figure 4a showing the gradual lengthening of PL decay which saturated at *k* = 0.7 μs^–1^, corresponding to a PL lifetime of τ ∼ 1.4 μs below 15 K. Crucially, this occurred at constant PLQY (Figure S23), indicating that the optical properties emerged from the band-edge excitonic fine structure comprised of a low-lying dark excitonic state (|D⟩) with total angular momentum *J* = 0 energetically separated by an optically active bright triplet state (|B⟩) with *J* = 1, as theoretically predicted using electron–hole exchange^47,59^ or atomistic arguments^60^ in the strong confinement regime. By assuming a Boltzmann distribution of excitons between the |D⟩ and |B⟩ states, we could model the transition between the slow and fast kinetics in a three-level scheme, by expressing the temperature dependence of the total decay rate as
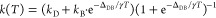
1where *k*_D_ and *k*_B_ are the radiative decay rates of the |D⟩ and |B⟩ states, respectively, γ is the Boltzmann constant, and Δ_DB_ is the dark–bright energy splitting.^61^ The model well reproduced the experimental data, returning a splitting energy of Δ_DB_ = 14.5 meV, in good agreement with the theoretical prediction for CsPbBr_3_ QDs of this size^62,63^ and with experimental values measured for other strongly confined nanocrystals^64,65^ also considering possible differences in the dielectric confinement term of the samples analyzed in the literature.

**Figure 4:**
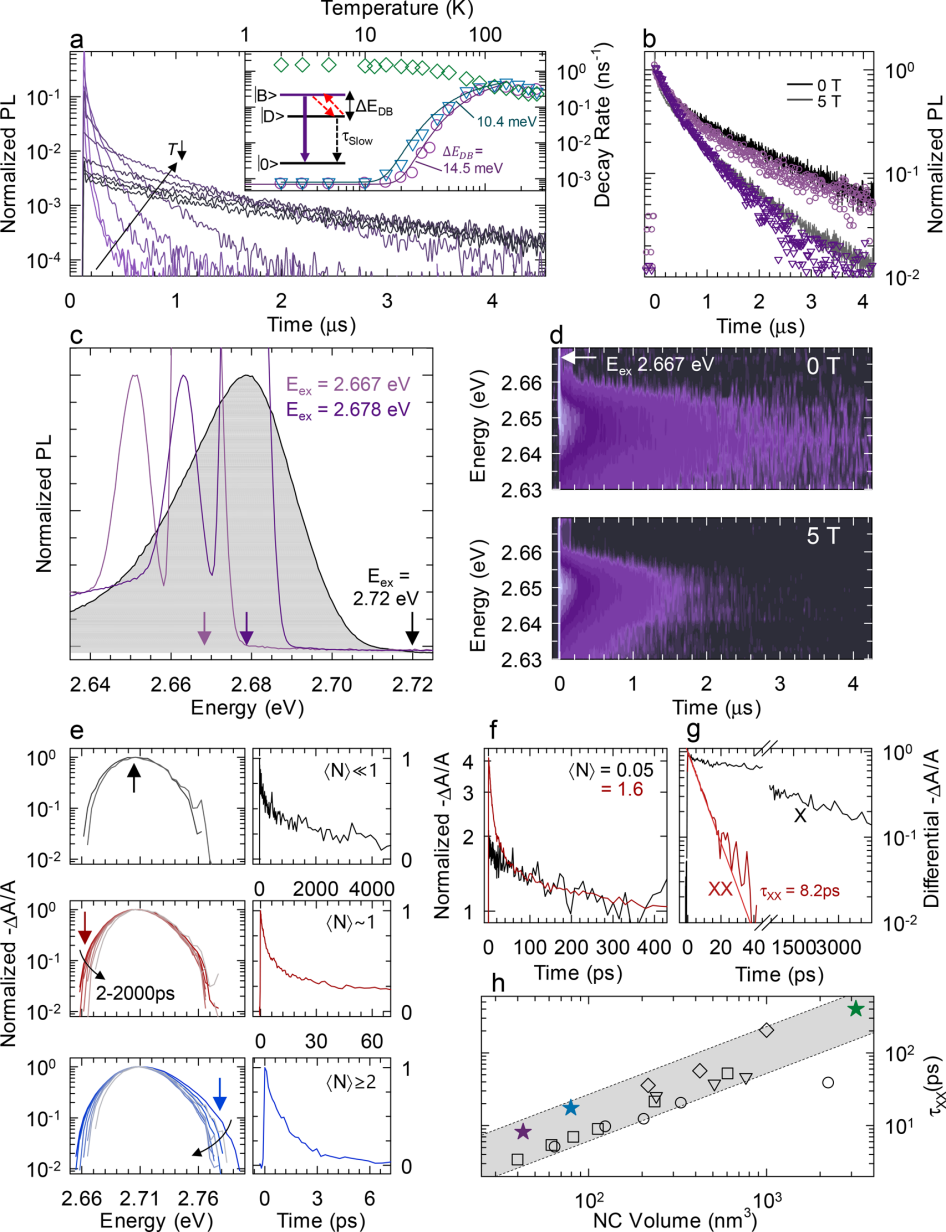
(a) Normalized PL decay traces in the temperature range 2–300 K for CsPbBr_3_ QDs with *d* = 3.5 nm. Inset: the extracted PL decay rates (circles) compared to the decay rates for *d* = 4.3 nm QDs (triangles) and *d* = 14.7 nm nanocrystals (diamonds). The solid lines are the fit with a 3-level model for the two smaller QDs. (b) Normalized PL decays at 5 K in the absence (black line) and in the presence (gray line) of a 5 T magnetic field. The circle and triangle data points represent the PL decay at 0 and 5 T, respectively, extracted from the FLN contour in ‘d’. (c) Normalized PL spectra collected in FLN mode at 5 K for different excitation energies as indicated in the figure. The shaded area corresponds to the ensemble PL spectrum excited under non-resonant conditions at 2.72 eV. (d) Time and spectrally resolved FLN decay curves collected at 5 K in the absence (top) and in the presence (bottom) of a 5 T magnetic field. (e) Transient absorption spectra (logarithmic scale) at increasing time (*t* = 2, 4, 6, 10, 20, 50, 500, 2000 ps) after the excitation pulse for progressively larger average exciton population ⟨*N*⟩ showing the emergence of bi- and multiexciton spectral contributions. The respective decay traces taken at the energies indicated in the figure are shown in the right-hand panels, highlighting gradually faster decay with increasing ⟨*N*⟩. (f) TA dynamics measured at 2.71 eV and normalized on the single exciton decay tail for ⟨*N*⟩ = 0.05 and 1.6. (g) Differential TA curve extracted from panel ‘f’ representing the biexciton dynamics. The solid line is the fit to a single-exponential decay. The single exciton TA trace is also reported in black for a direct comparison. (h) Measured biexciton lifetime (τ_XX_) of CsPbBr_3_ nanocrystals as a function of nanocrystal volume together with other values reported in the literature and adapted from ref (62) (squares), ref (66) (circles), ref (59) (triangles), and ref (55) (diamonds). The shaded area is a guide for the eye.

For a further confirmation of the obtained values, we also prepared 4.3 and 14.7 nm size CsPbBr_3_ QDs, using the standard hot-injection synthesis approaches (Figure S24). Increasing the QD size to *d* = 4.3 nm reduced Δ_DB_ to 10.4 meV. The same analyses, run on the much larger CsPbBr_3_ nanocrystals with *d* = 14.7 nm, with a size beyond the quantum confinement regime, revealed that the exciton fine structure is inverted with the lower-lying exciton state being a bright triplet, resulting in increasingly faster recombination dynamics with decreasing temperature (diamonds in the inset of Figure 4a). Both such findings are in accordance with theory,^47^ and with previous reports.

Further independent confirmation of the exciton fine structure of small QDs came from the study of PL decay in a magnetic field and from fluorescence line narrowing (FLN) measurements. Specifically, as shown in Figure 4b, the application of an external magnetic field induced mixing between the dark and bright exciton states, leading to a faster PL decay even at low temperature.^67,68^ The Δ_DB_ energy splitting can also be investigated directly from spectral measurements as the Stokes shift between the so-called zero-phonon line (ZPL), corresponding to the emission from the |D⟩ state without the assistance of phonons, and an ultranarrow excitation line.^69−71^ In QD ensembles, where the PL width is broadened by both homogeneous and inhomogeneous effects, respectively, due to thermal effects and particle size inhomogeneity, this is performed by operating in FLN configuration at cryogenic temperatures, that is, by exciting the sample at the low energy edge of the PL peak. Such resonant excitation conditions result in size selective excitation only of the large particle subset in the ensemble, thus allowing the intrinsic signatures of exciton fine structure to emerge. This method has been largely used for studying chalcogenide QDs^72^ but so far was never applied to halide perovskite QDs. Figure 4c shows the FLN spectra at 5 K together with the corresponding ensemble PL when excited at 2.72 eV, slightly above the 1S absorption edge. Notably, whereas the ensemble PL was the typical Gaussian peak due to QD size distribution, pumping on the red tail of the PL resulted in a sharp ZPL peak red-shifted from the excitation line by Δ_DB_ = 15 meV. In fact, as the QD excitation is due to optical coupling between the ground state and the optically accessible bright exciton, the observed Stokes shift of the ZPL provides direct access to the energy splitting which closely matches with the value obtained from the analysis of PL dynamics. In order to unambiguously ascribe the FLN peak to the ZPL, we further measured the time-resolved FLN spectra reported in Figure 4d, showing that the ZPL in fact exhibited the μs-long decay of the dark exciton measured in the ensemble mode. When an external magnetic field was applied, the time dynamics of ZPL accelerated identically to the ensemble kinetics (Figure 4b). This further confirms our assignment and adds a valuable experimental assessment of the exciton fine structure in the strong confinement regime.

Besides the effects on the fine structure of single excitons, for strongly confined QDs it is important to study the multiexcitonic photophysics, which are typically largely dominated by Auger-type non-radiative processes.^72^ To this end, we used fluence-dependent TA spectroscopy to extract the kinetic and spectral components of single-, bi-, and multiexcitonic states in our CsPbBr_3_ QDs. Figure 4e shows the normalized TA bleaching spectra of 3.5 nm sized CsPbBr_3_ QDs at increasing probe delays in the 2–2000 ps time interval and at progressively higher average exciton population ⟨*N*⟩, exploring the single-exciton (X), biexciton (XX), and multiexciton (MX) regimes. For ⟨*N*⟩ ∼ 0.05, when the biexciton population is negligible, the TA spectra exhibited a peak at 2.71 eV due to the filling of band-edge states which decayed in ∼2.5 ns, consistent with the PL decay measured at vanishingly low excitation power, together with an ∼1.3 ns minor initial contribution likely due to residual electron trapping, consistent with the measured PLQY = 60%. At higher fluences, the biexcitons emerged in the TA spectrum as a lower-energy shoulder—consistent with the attractive nature of the biexciton in CsPbBr_3_ nanocrystals^66,73^—which decayed rapidly with a characteristic time of ∼9 ps essentially dominated by Auger non-radiative processes. Increasing further the excitation fluences, the TA spectra showed an additional contribution on the high-energy side of the 1S bleach peak. This contribution decayed in approximately 1.4 ps and was attributed to higher order multiexcitons, consistent with previous observations on CsPbBr_3_ nanocrystals.^59,74^ The detailed analysis of the TA dynamics is presented in Figure 4f,g where the biexciton component was extracted according to the procedure introduced by Klimov et al.^75^ subtracting the contributions of lower order excitons. The measured biexciton decay time was τ_XX_ = 8.2 ps. Recent reports^55,62,66,76^ have shown the general agreement of CsPbBr_3_ nanocrystals with the universal volume-scaling law of τ_XX_, similarly to other nanocrystal compositions with both direct and indirect energy gaps.^77^ To put our data in perspective, in Figure 4h we report the biexciton lifetime of the CsPbBr_3_ QDs obtained from the transformation of (K_0.18_Cs_0.82_)_4_PbBr_6_ nanocrystals (*d* = 3.5 nm) together with two control samples produced by hot injection (*d* = 4.3, 14.7 nm), showing good agreement with the general volume scaling.

In summary, we demonstrated a facile approach to synthesize strongly quantum confined cubic-shaped CsPbBr_3_ QDs below 3.5 nm size. The method is based on a slow dissolution/recrystallization reaction of (K_0.18_Cs_0.82_)_4_PbBr_6_ nanocrystals upon reacting with PbBr_2_. The synthesis of (K_0.18_Cs_0.82_)_4_PbBr_6_ nanocrystals was performed through a K-exchange reaction on the as-synthesized Cs_4_PbBr_6_ nanocrystals. The K^+^ incorporation into Cs_4_PbBr_6_ nanocrystals occurs spontaneously at room temperature and stabilizes the crystal lattice, allowing the slowdown of the recrystallization reaction kinetics and thus the CsPbBr_3_ QD growth control. By varying the amount of (K_0.18_Cs_0.82_)_4_PbBr_6_ nanocrystals in the recrystallization reaction and thus the reaction time we can precisely tune the size and PL emission of the CsPbBr_3_ QDs in a range from ∼444 to 462 nm. The as-synthesized CsPbBr_3_ QDs show good long-term stability, narrow size distribution, and excellent optical properties, including tunable absorption and PL emission and with PLQY ∼ 60% (for 3.5 nm size). In-depth spectroscopic analysis provides the first investigation of the exciton fine structure using *cw* and time-resolved line-narrowing techniques in CsPbBr_3_ QDs and confirms the equivalence of these particles with those synthesized with more traditional methods, further expanding our knowledge of the photophysics of strongly confined lead halide perovskite QDs.

## Supporting Information Available

Detailed description of experimental methods and additional data including nanocrystal synthesis, TEM and STEM characterization, XRD and profile fitting, elemental mapping (EDS), elemental analysis (ICP), ^1^H NMR characterization, and computational methods (PDF)
