# Supplementary material for: Ultrasmall CsPbBr3 Blue Emissive Perovskite Quantum Dots Using K-Alloyed Cs4PbBr6 Nanocrystals as Precursors
Source: ACS Energy Lett. 2024 Apr 23;9(5):2367–77. doi: 10.1021/acsenergylett.4c00693 (PMC11450558; doi:10.1021/acsenergylett.4c00693)
Supplement: Supplementary file 1 — nz4c00693_si_001.pdf [file nz4c00693_si_001.pdf]

Supporting information

Ultrasmall $CsPbBr_3$ Blue Emissive Perovskite Quantum Dots using K-alloyed Cs_4PbBr_6 Nanocrystals as Precursors

Clara Otero-Martínez[1,2‡], Matteo L. Zaffalon[3‡], Yurii P. Ivanov[4], Nikolaos Livakas[2,5], Luca Goldoni[6], Giorgio Divitini[4], Sankalpa Bora[7], Gabriele Saleh[2], Francesco Meinardi[3], Andrea Fratelli[3], Sudip Chakraborty[7], Lakshminarayana Polavarapu[1], Sergio Brovelli[3], Liberato Manna[2]**

[1] *CINBIO, Department of Physical Chemistry, Materials Chemistry and Physics Group, Universidade de Vigo, Campus Universitario As Lagoas-Marcosende, 36310 Vigo, Spain*

[2] *Nanochemistry, Istituto Italiano di Tecnología, Via Morego 30, 16163 Genova, Italy*

[3] *Dipartimento di Scienza dei Materiali, Università degli Studi di Milano-Bicocca, Via R. Cozzi 55, 20125, Milano, Italy*

[4] *Electron Microscopy and Nanoscopy, Istituto Italiano di Tecnología, Via Morego 30, 16163, Genova, Italy*

[5] *Dipartimento di Chimica e Chimica Industriale, Università di Genova,16146 Genova, Italy*

[6] *Material Characterization Facility, Istituto Italiano di Tecnologia Via Morego 30, 16163 Genova, Italy*

[7] *Materials Theory for Energy Scavenging (MATES) Lab, Department of Physics, Harish-Chandra Research Institute (HRI), A C.I. of Homi Bhabha National Institute (HBNI), Chhatnag Road, Jhunsi, Prayagraj 211019, India*

‡ Equal Contribution

AUTHOR INFORMATION

Corresponding Author

*Liberato Manna - Nanochemistry, Istituto Italiano di Tecnologia, 16163 Genova, Italy; orcid.org/0000-0003-4386-7985; Email: liberato.manna@iit.it

*Sergio Brovelli - Dipartimento di Scienza dei Materiali, Università degli Studi di Milano-Bicocca, 20125 Milano, Italy; orcid.org/0000-0002-5993-855X; Email: sergio.brovelli@unimib.it

Materials and methods

Materials: Cesium carbonate (Cs_2CO_3, 99,9%), potassium acetate (CH_3COOK, > 98 %), lead bromide ($PbBr_2$, >98%), lead acetate trihydrate ($Pb(CH_3COO)_2 \cdot 3H_2O$, 99.99%), benzoyl bromide (C_6H_5COBr, 97%), octadecene ($C_{18}H_{36}$, 90%) oleic acid ($C_{18}H_{34}O_2$, 90%), oleylamine ($C_{18}H_{37}N$, 70%), toluene (C_7H_8, >99.8%), acetonitrile (CH_3CN, > 99.9 %), ethyl acetate ($C_4H_8O_2$), deuterated dimethyl sulfoxide-d$_6$ ($(CD_3)_2SO$,) and deuterated toluene-d$_8$ ($C_6D_5CD_3$, 99.95%) were purchased from Merck. All chemicals were used without further purification.

Precursor solutions preparation

$PbBr_2$ precursor solution 1 (Cs_4PbBr_6 synthesis). $PbBr_2$ (60 mg), 5 mL of octadecene, 1.5 mL of oleylamine and 200 μL of oleic acid were loaded in a 40 mL vial. The solution was heated-up to 120 ºC under stirring for 30 min. After this time, the salt was completely dissolved and the solution was cooled down.

Cs-OL solution (Cs_4PbBr_6 synthesis). Cs_2CO_3 (400 mg) and 8 mL of oleic acid were loaded in a 25 mL vial. The solution was heated up to 100 ºC under stirring and degassed under vacuum for 1 hour. After this time, the salt was completely dissolved solution was cooled down.

K-OL solution (cation exchange reaction). First, potassium acetate (250 mg) and 10 mL of oleic acid were loaded in a 25 mL vial to prepare a 0.25 M K-OL solution. The mixture was heated-up to 100 ºC under stirring and degassed under vacuum during 1 hour. After that, the salt was completely dissolved and the solution was cooled down. For the K-exchange reactions, 1 mL of the 0.25 M K-OL solution was diluted in 9 mL of toluene to prepare a ~ 25 mM K-OL solution.

$PbBr_2$ precursor solution 2 ($CsPbBr_3$ QDs synthesis). $PbBr_2$ (367 mg), 5 mL of toluene, 2.5 mL of oleylamine and 2.5 mL of oleic acid were loaded in a 25 mL vial. The solution was heated up to 120 ºC under stirring for 1 hour. After this time, the salt was completely dissolved and the solution was cooled down.

Pb-Cs-OL stock solution (large $CsPbBr_3$ nanocrystals synthesis). Lead acetate trihydrate (760 mg), Cs_2CO_3 (160 mg) and oleic acid (15 mL) were loaded in a 25 mL vial. The solution was heated up to 100 ºC under stirring and degassed for 1 hour. After this time, the precursors salts were completely dissolved and the solution was cooled down.

DDA stock solution (large $CsPbBr_3$ nanocrystals synthesis). Didodecylamine (4.43 g) and toluene (10 mL) were loaded in a 25 mL vial. To dissolve the salt, the mixture was sonicated for 10 min at 50 ºC in a sonication bath.

Nanocrystal synthesis

Cs_4PbBr_6 nanocrystals synthesis. The synthesis of Cs_4PbBr_6 nanocrystals was performed accordingly a previously reported method[1] with slight modifications. Briefly, the **$PbBr_2$ precursor solution 1** previously prepared was heated up to 80 ºC in a 20 mL vial under stirring and N_2 atmosphere. When the solution reached the desired temperature, 0.75 mL of the Cs-OL solution was swiftly injected. After ~ 30 s, the solution became white-turbid indicating the formation of the Cs_4PbBr_6 nanocrystals and the reaction was quenched in an ice-bath. The solution was purified by centrifugation (6000 rpm, 5 min) without use of antisolvents and the precipitated was collected and redispersed in 1 mL of toluene.

$(K_{0.18}Cs_{0.82})_4PbBr_6$ nanocrystals synthesis. The synthesis of $(K_{0.18}Cs_{0.82})_4PbBr_6$ nanocrystals was performed by 3 cycles of cation exchange reaction in air conditions and room temperature. In each cycle, 1 mL of the 25 mM K-OL solution in toluene was added to the as-synthesized Cs_4PbBr_6 nanocrystals solution in toluene in a 7 mL vial. Then, 5 mL of ethyl acetate were added to the colloidal solution to induce the precipitation of the nanocrystals. The solution was centrifuged (6000 rpm, 5 min) and the precipitate was collected and redispersed in a 1 mL oleylamine & oleic acid ligand solution in toluene of 2.5 mM concentration to recover the losses of ligands during the washing with antisolvent. This process was repeated 2 more times to complete the 3 cycles of K-exchange.

$CsPbBr_3$ QDs synthesis. The synthesis of $CsPbBr_3$ QDs was performed by a dissolution-recrystallization reaction of $(K_{0.18}Cs_{0.82})_4PbBr_6$ nanocrystals when reacted with $PbBr_2$. Briefly, 5

mL of toluene and the desired volume of $(K_{0.18}Cs_{0.82})_4PbBr_6$ nanocrystals were loaded in a 7 ml vial. The solution was heated up to 100 °C under stirring and N_2 atmosphere. When the solution reached the desired temperature, 1 mL of the **$PbBr_2$ precursor solution 2** was swiftly injected. After the corresponding reaction time, the solution was quenched in an ice-water bath. The specific volumes of $(K_{0.18}Cs_{0.82})_4PbBr_6$ nanocrystals and the corresponding reaction time are described in **Table S1**.

$CsPbBr_3$ QDs were purified by adding subsequently 0.5 mL of EtOAc and 0.5 mL of acetonitrile to 1 mL of crude solution. After the addition of the solvents the crude solution became turbid, meaning that the QDs lost the colloidal stability and the solution was quickly centrifuged (4000 rpm, 2 min). The supernatant was discarded and the precipitate was redispersed in 1 mL of toluene.

Table S1. Reaction times and $(K_{0.18}Cs_{0.82})_4PbBr_6$ nanocrystals volumes employed to obtained $CsPbBr_3$ QDs with their corresponding excitonic peak.

Reaction time (min)	Volume $(K_{0.18}Cs_{0.82})_4PbBr_6$ nanocrystals (mL)	Excitonic peak (nm)
5	0.05	420.0
10	0.1	425.7
15	0.15	430.7
30	0.25	436.2
50	0.35	439.7
100	0.45	442.3
135	0.5	448.0
160	0.55	452.5

4.3 and 14.7 nm $CsPbBr_3$ nanocrystals synthesis. The synthesis of $CsPbBr_3$ nanocrystals was performed according previously reported methods with slight modifications.[2] Briefly, octadecene (9 mL), **Pb-Cs-OL sock solution** (1.5 mL) and **DDA stock solution** (1.5 mL) are loaded in a 25 mL vial and heated up under N_2 and stirring to 50 and 160 °C to obtain 4.3 and 15 nm size respectively. Then, a solution of benzoyl bromide (50 µL) in toluene (500 µL) was

swiftly injected. After 1 min, the reaction was quenched in an ice-water bath. When the reaction was cooled down, 200 µL of oleylamine was added to the crude solution to obtain the OLAm & OL surface capping composition. The colour change to a bright green indicates the oleylammonium binding to the surface. The solutions were then purified by centrifugation (6000 rpm, 5 min) after adding 6 mL of EtOAc to 3 mL of the crude solution. Finally, the precipitate obtained was redispersed in 5 mL of decane for the spectroscopy measurements.

NMR characterization

NMR experiment were acquired at 298 K, on a Bruker Avance III 600 MHz (600.13 MHz) spectrometer, fit with a 5 mm QCI cryoprobe. Prior to the acquisition, matching and tuning and, line shape resolution, were automatically adjusted. The 90° pulse was calculated on each tube by using Bruker's automatic routines.[3] ^{1}H NMR spectra in toluene-d_8 were performed without steady scan, with 256 scans and 65536 points of digitalization, an inter pulses delay of 30 s, at a fixed receiver gain (18), on a spectral width of 20.83 ppm centered at 6.18 ppm.

For the *^{1}H quantitative* NMR spectra in DMSO-d_6 used for concentration measurement with PULCON method, identical acquisition parameters were employed except for the number of transients (64).

Prior to the Fourier transform an exponential smoothing function equivalent to 0.3Hz was applied to FIDs. Spectra were manually phased and automatically baseline corrected.

Sample preparation. For the NMR characterization, Cs_4PbBr_6 and $(K_{0.18}Cs_{0.82})_4PbBr_6$ nanocrystals solutions in toluene (1 mL) were purified with EtOAc (6 mL) by centrifugation (6000 rpm, 5 min). Then, the precipitates were dried with a N_2 flow and redispersed in deuterated toluene (500 µL). The colloidal solution was loaded into a 5 mm disposable sampleJet tubes (Bruker). For the ligand quantification, the colloidal solutions already characterized in deuterated toluene, where dried using a N_2 flow and the resulting material was dissolved in deuterated DMSO (200 µL) and loaded into a 3 mm disposable sampleJet tube.

Ligand quantification. The concentration of both ligands ([OLAm & OL]) was quantified using *quantitative* NMR and PULCON (PUlse Length-based CONcentration determination) method[4], comparing the integrated peak intensity of ligand signals to that of standard external solution of dimethylsulfone (10 mM, TraceCERT®) in DMSO-d_6, after normalizing each signal to the number of ^{1}H resonances generating the peak. The peaks used for quantification are the pseudo triplet at 2.75 ppm for OLAm and triplet at 2.1 ppm for OL.

Total ligand concentration = [OLAm + OL] = $[OLAm]_{\text{measured by PULCON}} + [OL]_{\text{measured by PULCON}}$ ***SEq. 1***

Inductively Coupled Plasma−Optical Emission Spectroscopy (ICP-OES).

After the ligand quantification, the Pb concentration of the nanocrystals dissolved in deuterated DMSO-d_6 was obtained by ICP-OES on an aiCAP 6000 spectrometer (Thermo Scientific). Prior the measurement, 50 µL of the DMSO – d_6 sample were diluted to 10 mL in an aqueous solution of aqua regia (1:10) and subjected to an acid digestion overnight.

Ligand density

The ligand density of the samples was obtained by calculating the ratio of the ligand concentration and nanocrystal surface in the DMSO-d_6 solution:

$$Ligand\ density = \frac{[Ligand](unit/mL)_{PULCON}}{Total\ NC\ surface\ (nm^2/mL)}$$ ***SEq. 2***

The total nanocrystal surface was determined through the Pb concentration in the DMSO-d_6 sample (measured by ICP) and the size distribution of the nanocrystals considering a unit cell volume of 2.82 and 2.78 nm^3 for Cs_4PbBr_6 and $(K_{0.18}Cs_{0.82})_4PbBr_6$ nanocrystals respectively.

Computational Methodology

In order to substitute K-atoms in the Cs_4PbBr_6 lattice we used the Site-Occupation Disorder (SOD) program.[5] Subsequently, we systematically determined various electronic properties, including the density of states, band structure, and optical spectra for the studied configurations. We conducted comprehensive electronic structure calculations based on first principles using the density functional theory (DFT) framework [5, 6] within the Vienna ab-initio simulation package (VASP).[7] Throughout the geometry optimization and electronic structure calculations, we considered the projected-augmented-wave (PAW) formalism[8] and employed the generalized gradient approximation (GGA) for the exchange-correlation functional.[9] For the bulk geometry optimization, we utilized a plane wave basis set with a 400 eV energy cutoff and a 4x4x3 Monkhorst Pack k-points sampling scheme.[10] We ensured that all considered systems were fully relaxed according to the minimum-energy criteria, continuing the optimization until the Hellman-Feynman force dropped below 0.02 eV/Å. For density of states calculation, we took a denser grid k-points using 9x9x6 Monkhorst pack scheme and specified the number of energy

grid points to 3000 while keeping the other parameters same. The band structure and optical spectra calculations were done using the same parameters but with 4x4x3 k-points.

Optical characterization

UV-Vis absorption spectra were carried out using a Varian Cary 300 UV−Vis absorption spectrophotometer (Agilent). The spectra were collected by diluting 50 µL of the sample in toluene in 2.5 mL of hexane in order to collect the spectra until ≈ 250 nm and analyse the features of Cs_4PbBr_6 and $(K_{0.18}Cs_{0.82})_4PbBr_6$ nanocrystals. Photoluminescence spectra were obtained on a Varian Cary Eclipse Spectrophotometer (Agilent) using λ_{ex}= 350 nm ($CsPbBr_3$ QDs) and 270 nm (Cs_4PbBr_6 and $(K_{0.18}Cs_{0.82})_4PbBr_6$ nanocrystals). Time resolved photoluminescence spectra at room temperature and PL quantum yield measurements were obtained using an Edinburgh FLS900 fluorescence spectrophotometer. PL decay traces were measured with a pulsed laser diode (λ_{ex}= 375, pulse width = 50 ps). Quantum yield measurements were acquired using a calibrated integrating sphere with λ_{ex} = 350 nm for all of the measurements. All solutions were diluted to an optical density of 0.1 - 0.2 at the excitation wavelength in order to minimize the reabsorption of the fluorophore. Quartz cuvettes with an optical path length of 1 cm were used for all optical analyses.

Temperature-dependent and time-resolved PL measurements were performed on QD thin films drop-cast on quartz substrates and mounted in a closed-circuit He cryostat with optical access and equipped with superconducting coils for magnetic field generation. The excitation source was a pulsed laser at 3.06 eV (405 nm, ∼70 ps pulses) and the emitted light was dispersed with a spectrometer and detected with a charge-coupled device for *cw* measurements and with a photomultiplier tube coupled with a time-correlated single-photon counting unit for time-resolved PL measurements (time resolution ∼400 ps).

For FLN measurements, a spectrally narrowed (~0.5 nm full width at half maximum) fs-pulsed laser was generated by coupling the output of a tunable optical parametric amplifier to a 1/3 m double-grating Gemini monochromator. The emitted PL was collected with a Horiba Scientific Triax 180 1/2 m spectrograph and detected with a cooled charge-coupled device.

Ultrafast transient absorption spectroscopy measurements were performed on a Helios TA spectrometer from Ultrafast Systems. The laser source was a 10 W Hyperion amplified laser operating at 1.875 kHz and producing ~260 fs pulses at 1030 nm, coupled to an independently tunable APOLLO-Y optical parametric amplifier from the same supplier that produced the

excitation pulses at 3.1 eV. After passing the pump beam through a synchronous chopper phase-locked to the pulse train (0.937 kHz, blocking every second pump pulse), the pump fluence on the sample was modulated from 13 μJ cm^{-2} to 1700 μJ cm^{-2}. The probe beam was a white light supercontinuum.

Elemental analysis

Scanning electron microscopy (SEM) was performed on a HRSEM JEOL JSM-7500LA microscope with a cold field-emission gun (FEG), operating at acceleration voltage of 15 kV. The elemental composition of the samples was obtained by energy-dispersive spectroscopy (EDS, Oxford instrument, X-Max, 80 mm2) operating at 8 mm working distance, acceleration voltage of 15 kV, and 15 sweep count.

Transmission electron microscopy (TEM) images were obtained with a JEOL JEM 1011 transmission electron microscope operating at an acceleration voltage of 100 kV. High-resolution scanning transmission electron microscopy (HR STEM) images were acquired on a probe-corrected ThermoFisher Spectra 30-300 STEM operated at 300 kV. Images were acquired on a High-Angle annular Dark Field (HAADF) detector with a very small beam current of around 10 pA to avoid a beam damages of a few nanometres lead halide perovskite nanocrystals. Convergence angle was set to 25 mrad, it corresponds to sub angstrom electron beam. Compositional maps were acquired using Velox, with a probe current of ~150 pA and rapid rastered scanning. The Energy-Dispersive X-Ray (EDS) signal was acquired on a Dual-X system for a total acquisition angle of 1.76 Sr.

X-ray Powder Diffraction and profile fitting.

X-ray powder diffraction measurements were performed on a PANanalytical Empyrean X-ray diffractometer, equipped with a 1.8 kW Cu Kα ceramic anode and a PIXcel3D 2 × 2 area detector, operating at 45 kV and 40 mA. Nanocrystals' dispersions were mixed with fumed silica and dried to minimize the preferential orientation. Finally, the powder was placed on a zero-diffraction silicon substrate to perform the measurements. X-ray powder diffraction patterns were refined through Rietveld method employing Fullprof Suite software. The analysis included background extraction followed by a systematic refinement of various parameters involving scale factor, unit cell, instrumental and profile parameters (taking into account shape and asymmetry). Likewise, factors such as atomic coordinates, occupancy and thermal expansion were refined.

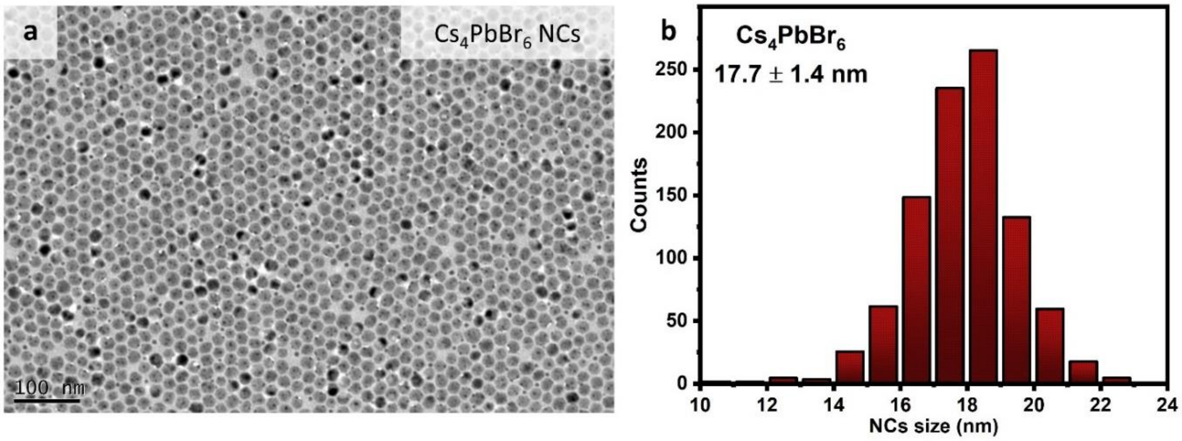

Figure S1. (a) TEM images of Cs_4PbBr_6 nanocrystals and (b) histogram obtained after the analysis of the corresponding TEM image. The estimated average size was 17.7 ± 1.4 nm.

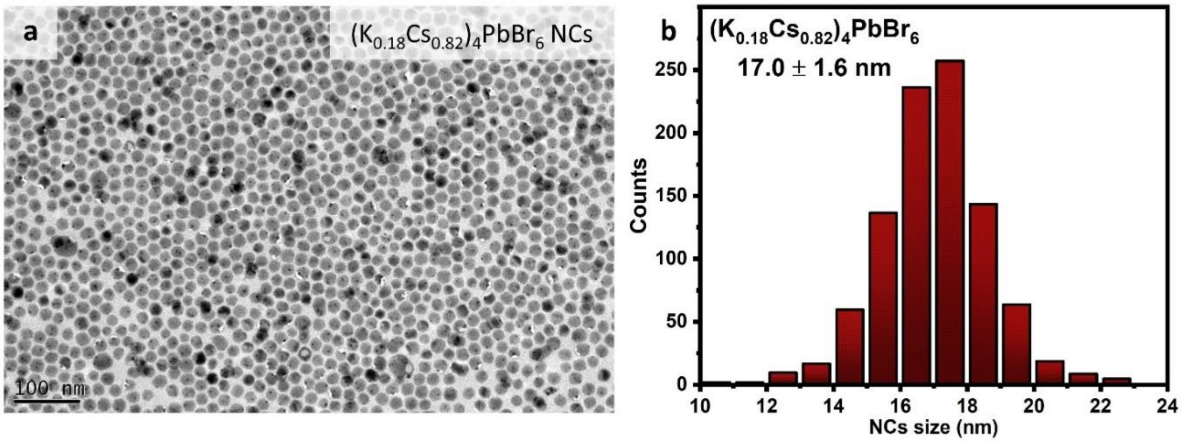

Figure S2. (a) TEM images of $(K_xCs_{1-x})_4PbBr_6$ nanocrystals obtained by cation-exchange reaction and (b) histogram obtained after the analysis of the corresponding TEM image. The estimated average size was 17.0 ± 1.6 nm.

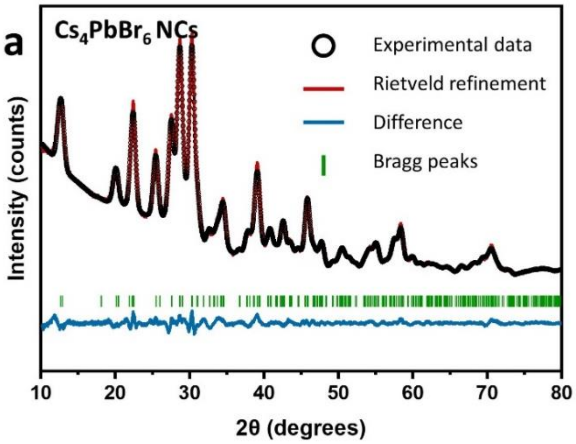

Cs_4PbBr_6 Nanocrystals	
Space group: R -3 c (#167-1)	
a = 13.71910 Å	α = 90.000 °
b = 13.71910 Å	β = 90.000 °
c = 17.30200 Å	γ = 120.000 °
V = 2820.1888 $Å^3$	
χ^2= 6.5828	

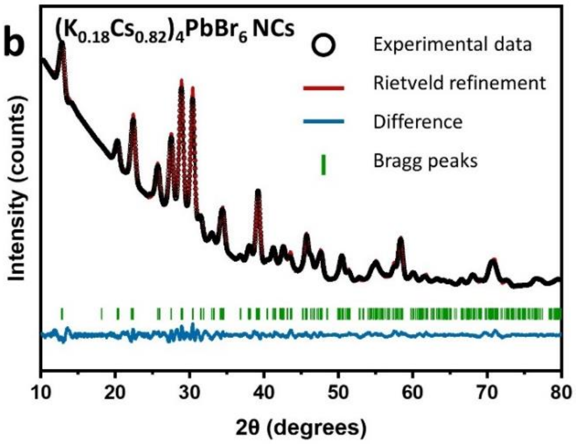

$(K_{0.18}Cs_{0.82})_4PbBr_6$ Nanocrystals	
Space group: R -3 c (#167-1)	
a = 13.74600 Å	α = 90.000 °
b = 13.74600 Å	β = 90.000 °
c = 17.05020 Å	γ = 120.000 °
V = 2790.0552 $Å^3$	
χ^2= 6.6460	

Figure S3. Structural characterization. Rietveld refinement of XRD diffraction patterns of Cs_4PbBr_6 nanocrystals (a) and K-doped $(K_{0.18}Cs_{0.82})_4PbBr_6$ nanocrystals (b). The black circles and red lines are the experimental and the calculated patterns. The lower line (blue) represents the residuals of the fit, while the vertical bars (green) correspond to the calculated positions of Bragg peaks. Refined parameters of the two structural models are presented in the corresponding tables.

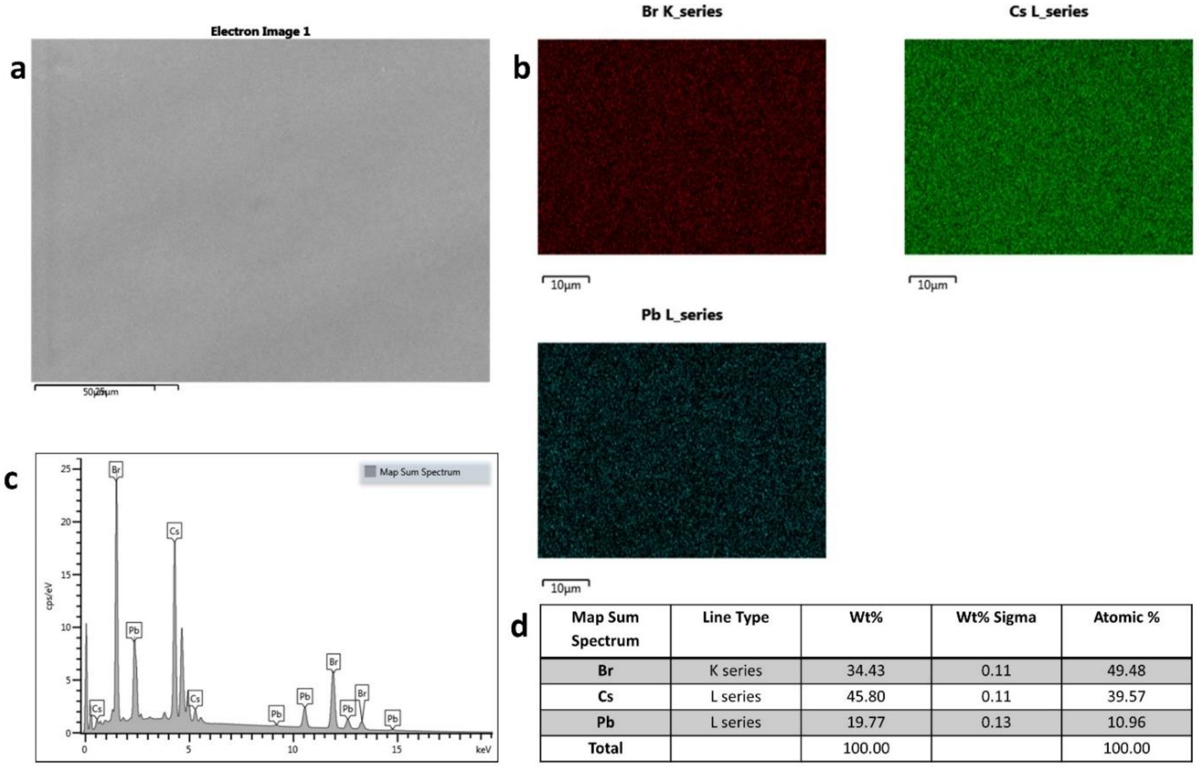

Map Sum Spectrum	Line Type	Wt%	Wt% Sigma	Atomic %
Br	K series	34.43	0.11	49.48
Cs	L series	45.80	0.11	39.57
Pb	L series	19.77	0.13	10.96
Total		100.00		100.00

Figure S4. a) SEM image of a Cs_4PbBr_6 nanocrystals film. b) EDS elemental images of Br, Cs and Pb. (c) EDS spectrum of the Cs_4PbBr_6 nanocrystals film from (a). (d) Summary of the elemental analysis obtained from the EDS spectrum expressed in atomic percentage (%). The scale bar is 50 µm in in (a) and 10 µm in (b).

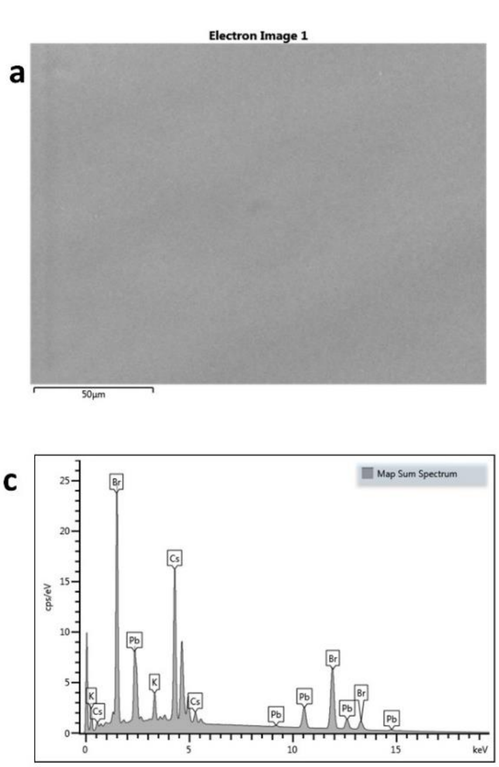

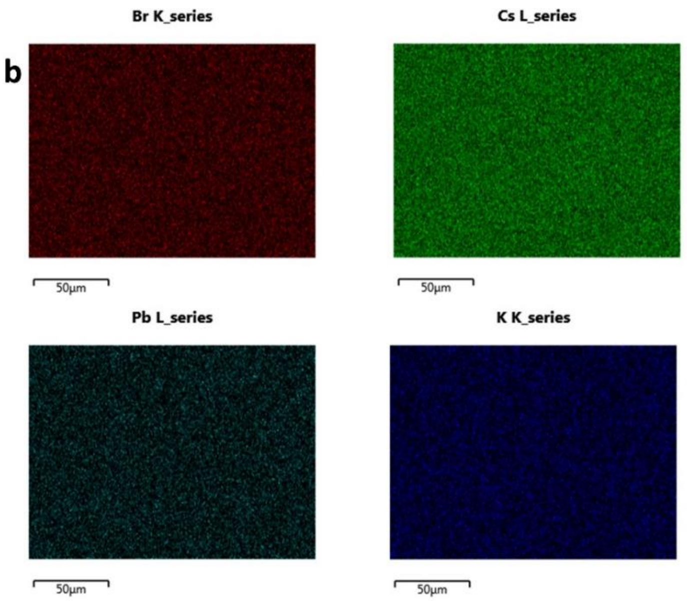

d

Map Sum Spectrum	Line Type	Wt%	Wt% Sigma	Atomic %
K	K series	2.59	0.03	7.14
Br	K series	36.81	0.12	49.64
Cs	L series	40.24	0.11	32.63
Pb	L series	20.36	0.13	10.59
Total		100.00		100.00

Figure S5. a) SEM image of a $(K_xCs_{1-x})_4PbBr_6$ nanocrystals film. b) EDS elemental images of Br, Cs, Pb and K. (c) EDS spectrum of the $(K_xCs_{1-x})_4PbBr_6$ nanocrystals film from (a). (d) Summary of the elemental analysis obtained from the EDS spectrum expressed in atomic percentage (%). The scale bar is 50 µm in all the cases.

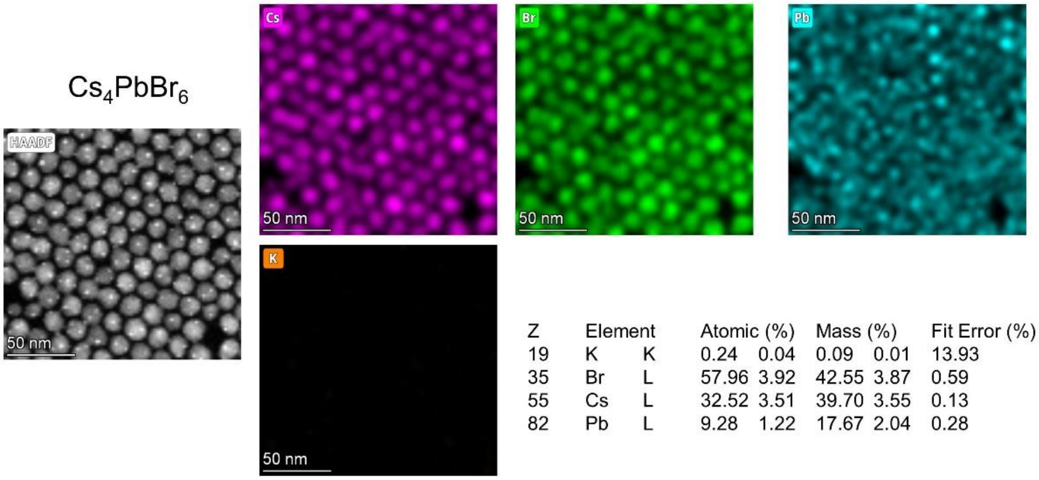

Figure S6. a) HAADF-STEM image of Cs_4PbBr_6 nanocrystals and corresponding EDS elemental images of Cs, Br, Pb and K.

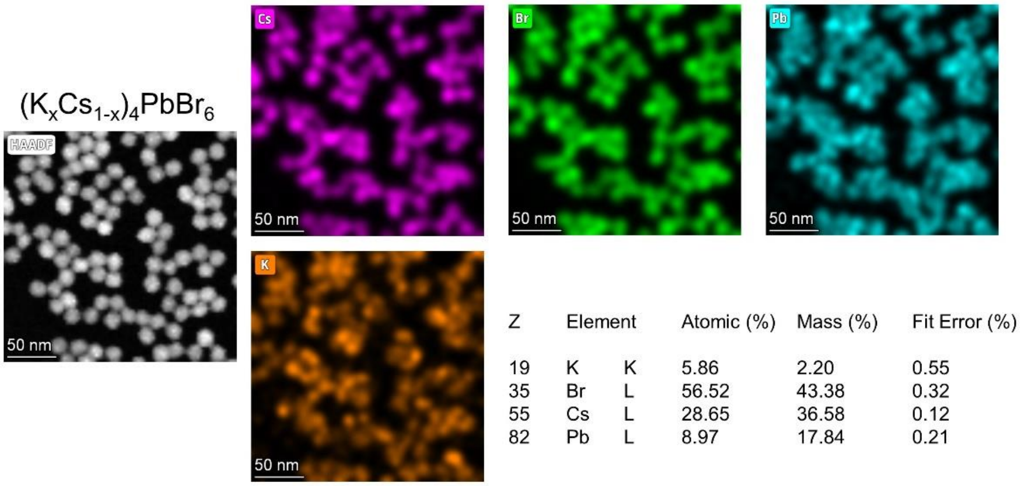

Figure S7. a) HAADF-STEM image of $(K_xCs_{1-x})_4PbBr_6$ nanocrystals and corresponding EDS elemental images of Cs, Br, Pb and K.

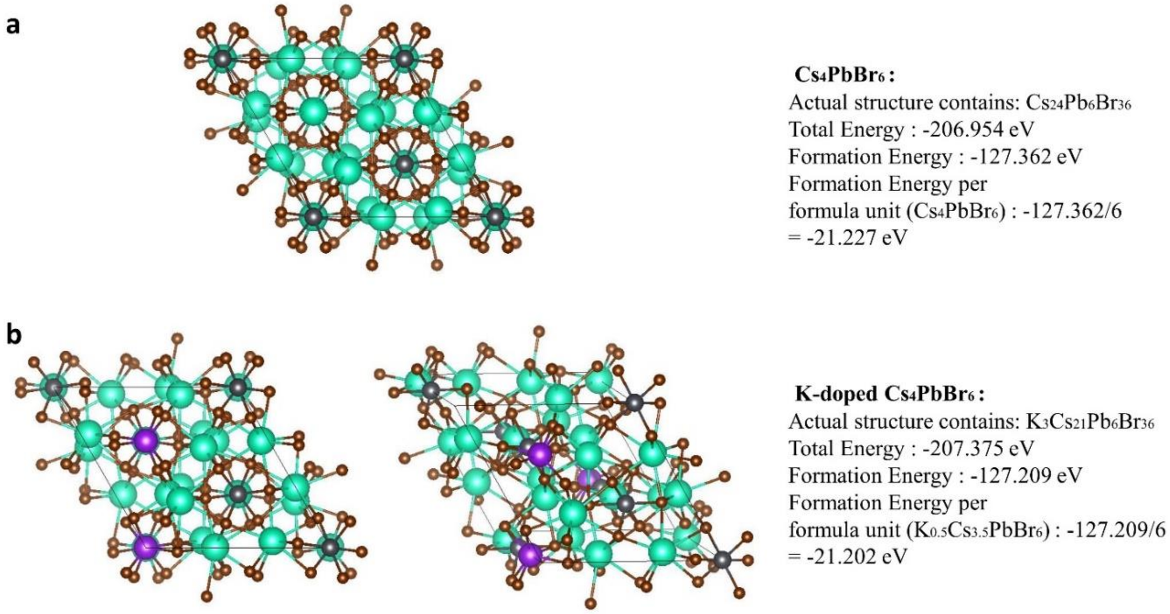

Figure S8. Crystal structures and corresponding energies of Cs_4PbBr_6 (a) and $(K_{0.125}Cs_{0.875})_4PbBr_6$ (b).

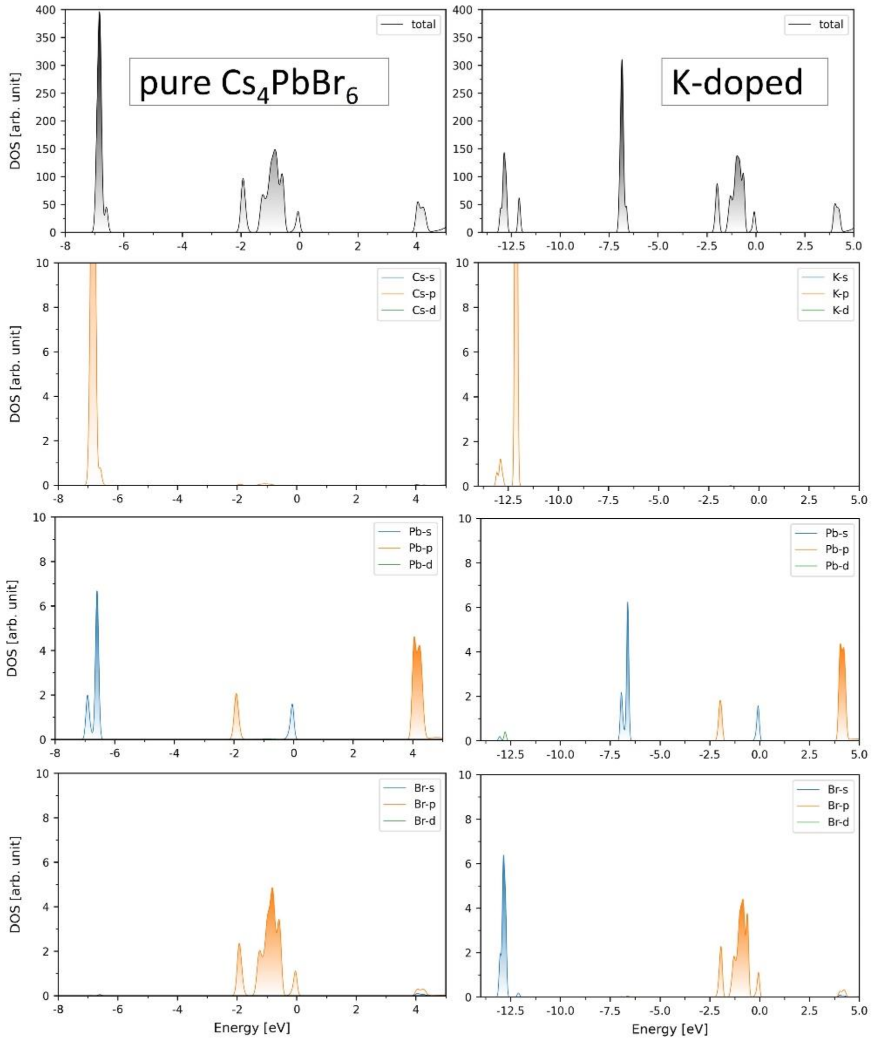

Figure S9. Density of states (DOS) for pure (left) and K-doped (right) Cs_4PbBr_6 (unit cell formulas: $Cs_{24}Pb_6Br_{36}$ and $K_3Cs_{21}Pb_6Br_{36}$, respectively). The top panels show the total DOS, while those below the atomic orbital contributions of each atom type (Cs for K-doped not shown for sake of space). For projected DOS, each panel shows 2 atoms (2 is the maximum number of symmetry-independent atoms among all atom types in the undoped compound), so that the DOS magnitudes are directly comparable.

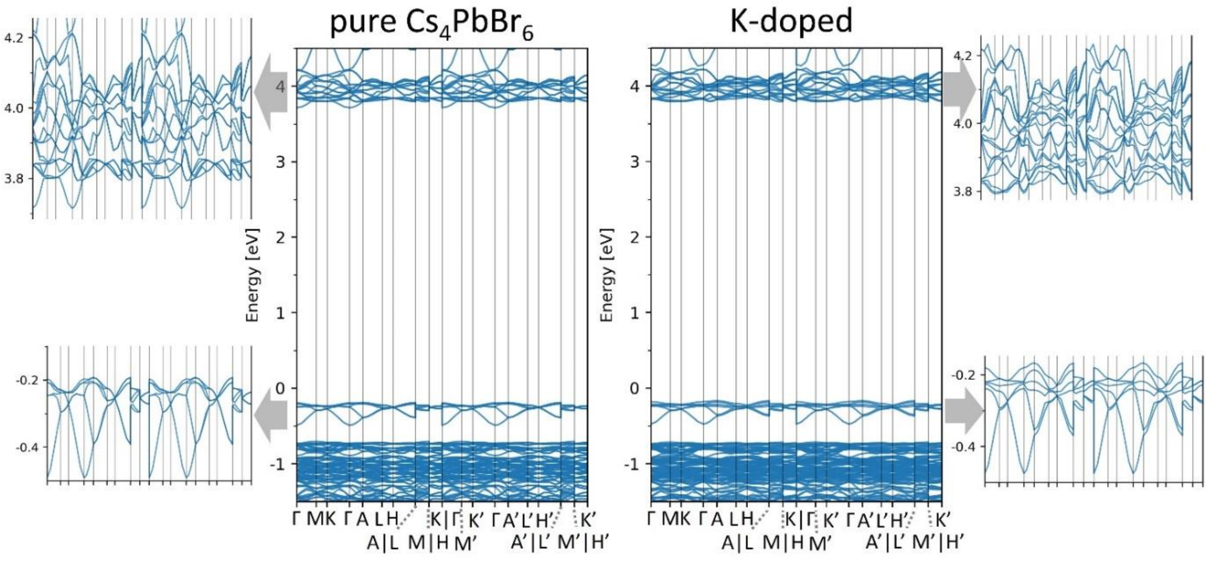

Figure S10. Band structure of pure and K-doped Cs_4PbBr_6. Enlargements of the valence and conduction bands are shows beside each plot, showing the indirect character of the band gap. Note that for both plots we adopted the reciprocal space path of the K-doped compound, to allow a direct comparison. The latter, indeed, is less symmetric (space group P321 *vs.* R-3c of the undoped compound) and thus requires a more extended reciprocal space path.

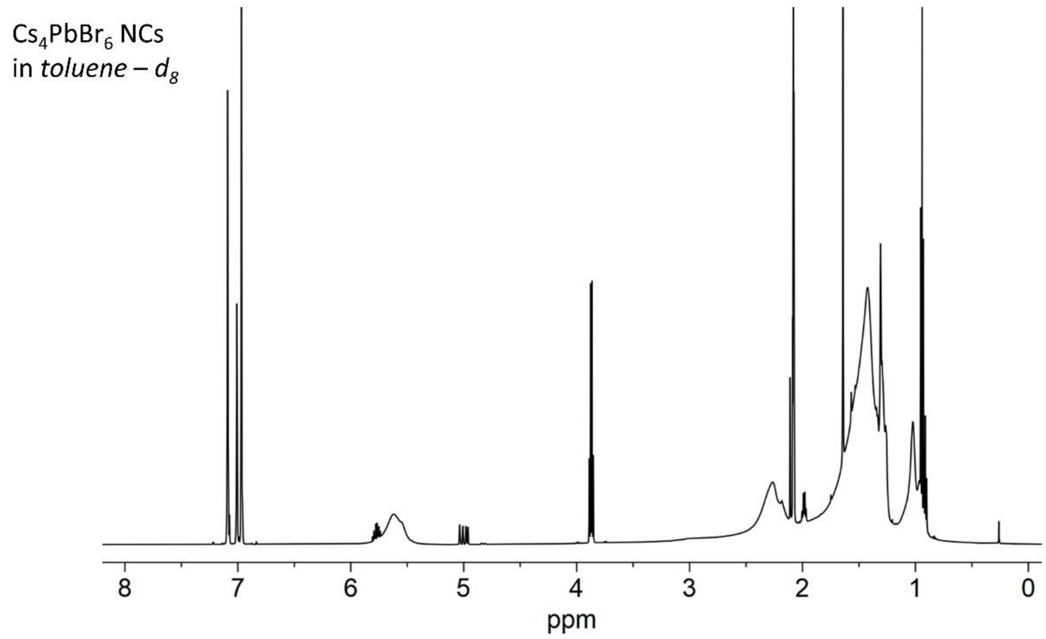

Figure S11. ^{1}H NMR spectrum of Cs_4PbBr_6 in toluene-d_8.

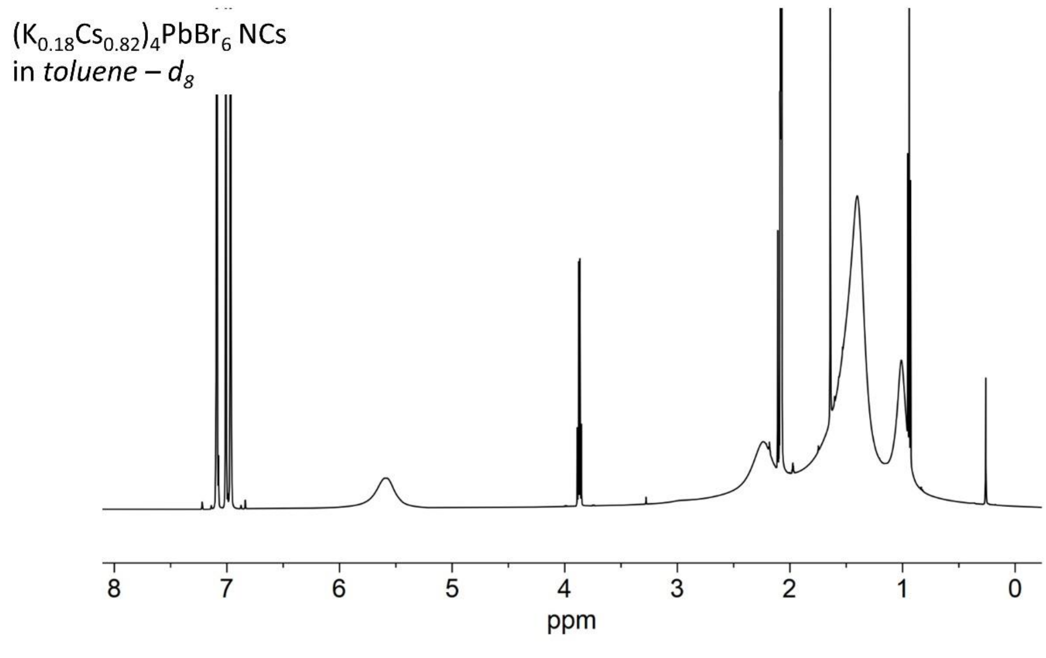

Figure S12. ^{1}H NMR spectrum of $(K_{0.18}Cs_{0.82})_4PbBr_6$ in toluene-d_8.

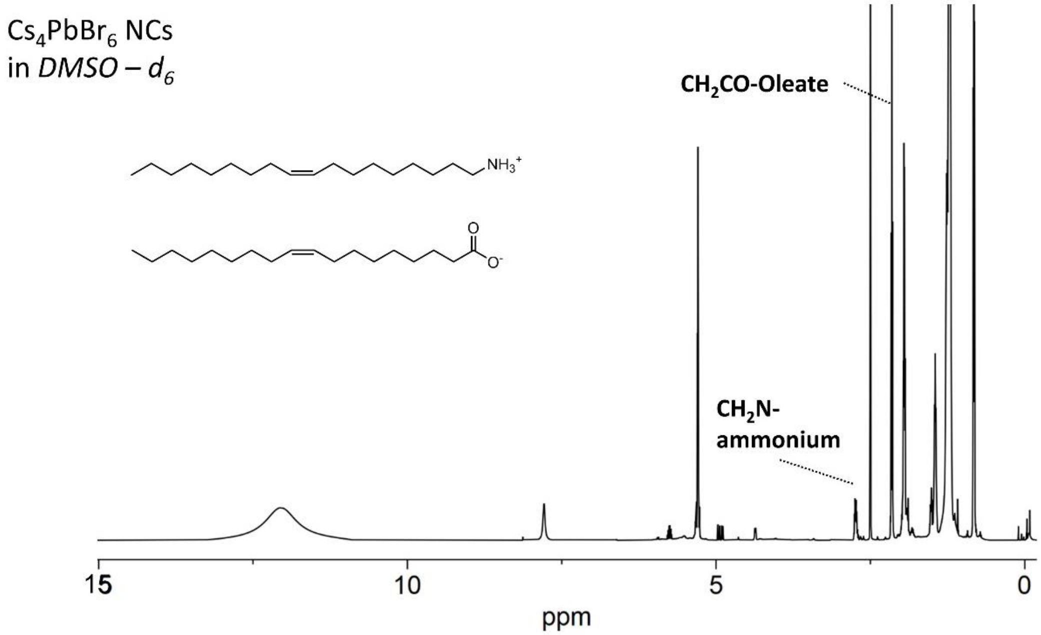

Figure S13. ^{1}H NMR quantitative spectrum of Cs_4PbBr_6 in DMSO-d_6 + 5 μL (2.5% v/v) of TFA. Peaks used for quantification are shown.

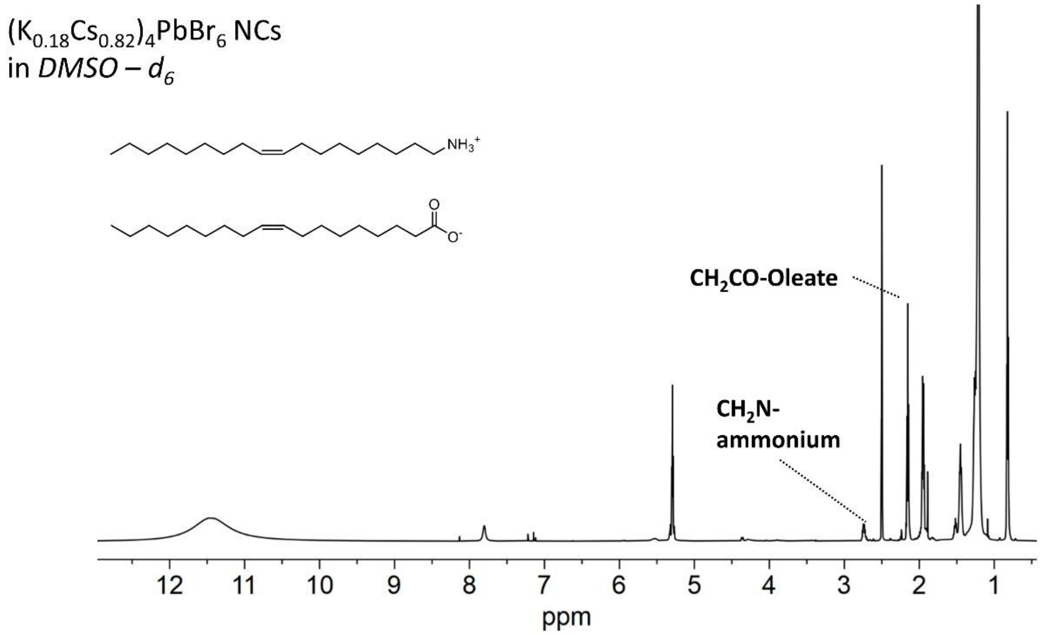

Figure S14. ^{1}H NMR quantitative spectrum of $(K_{0.18}Cs_{0.82})_4PbBr_6$ in DMSO-d_6 + 5 μL (2.5% v/v) of trifluoro acetic acid (TFA). Peaks used for quantification are shown.

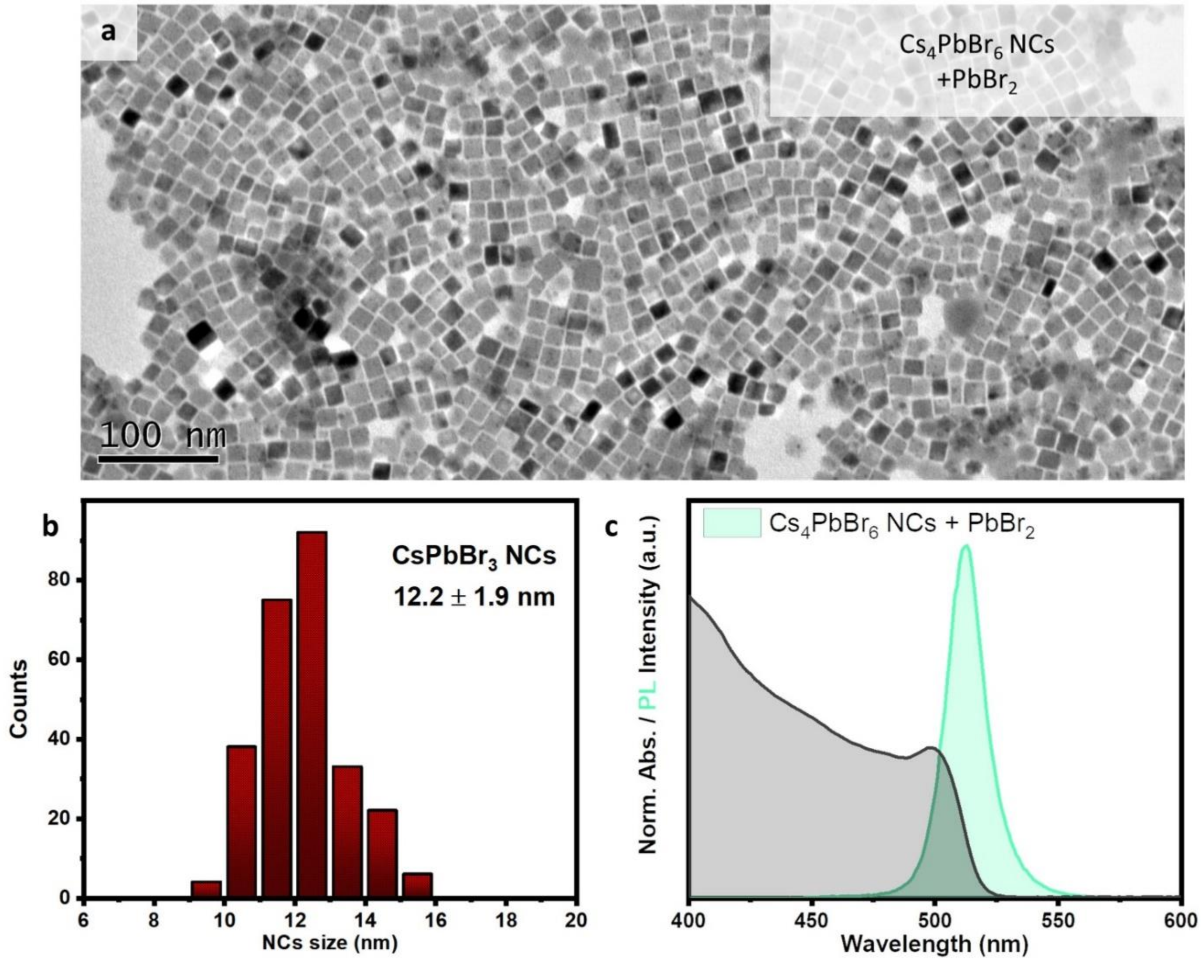

Figure S15. (a) TEM image of $CsPbBr_3$ nanocrystals obtained by the reaction of Cs_4PbBr_6 nanocrystals with $PbBr_2$. (b) Histogram obtained after the analysis of the corresponding TEM image. The estimated average size was 12.2 ± 1.9 nm.

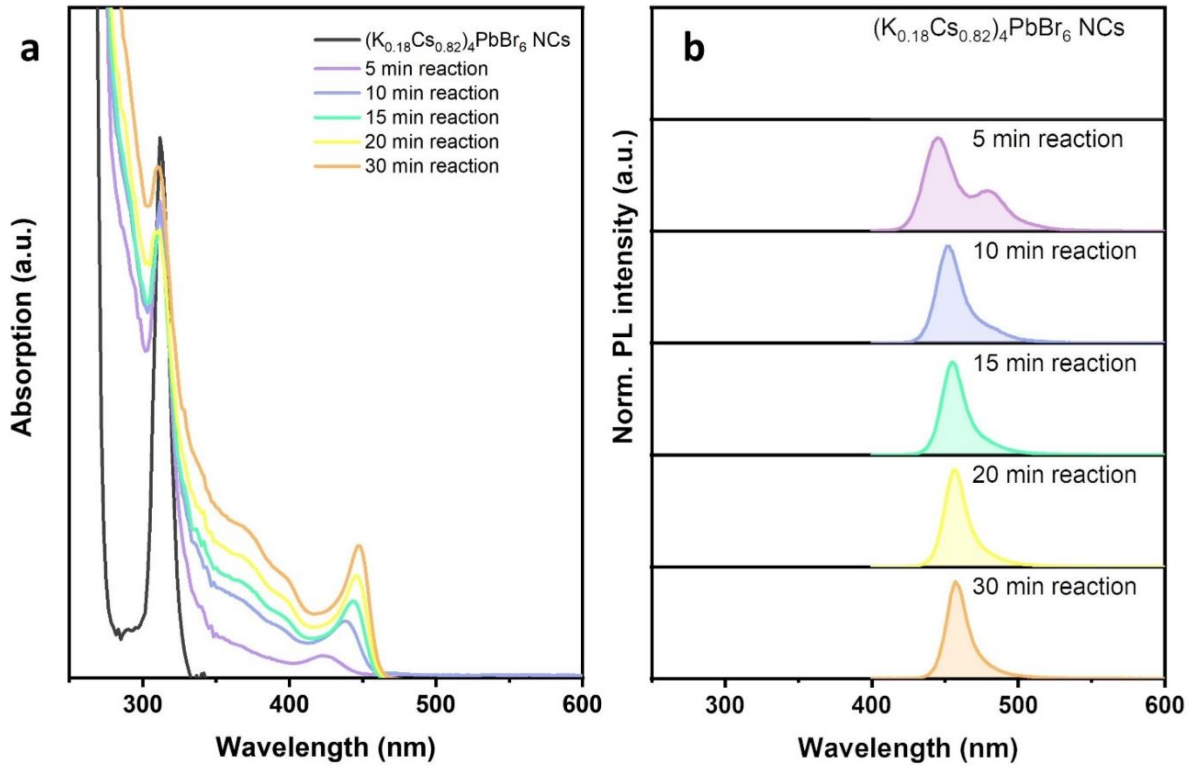

Figure S16. Absorption spectra evolution (a) and normalized PL spectra (b) of the $CsPbBr_3$ QDs at the early stages of the reaction.

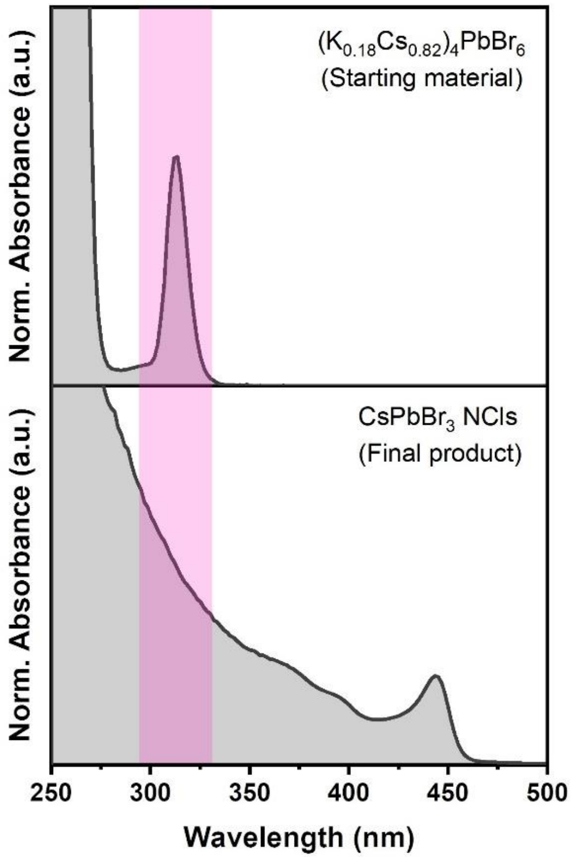

Figure S17. Normalized absorption spectra of the starting material ($(K_{0.18}Cs_{0.82})_4PbBr_6$ nanocrystals) in the top panel and the final product ($CsPbBr_3$ QDs) in the bottom panel. The pink mark indicates the position of the excitonic peak of $(K_{0.18}Cs_{0.82})_4PbBr_6$ nanocrystals that we used as reference to follow the reaction and whose disappearance indicates the end of the reaction.

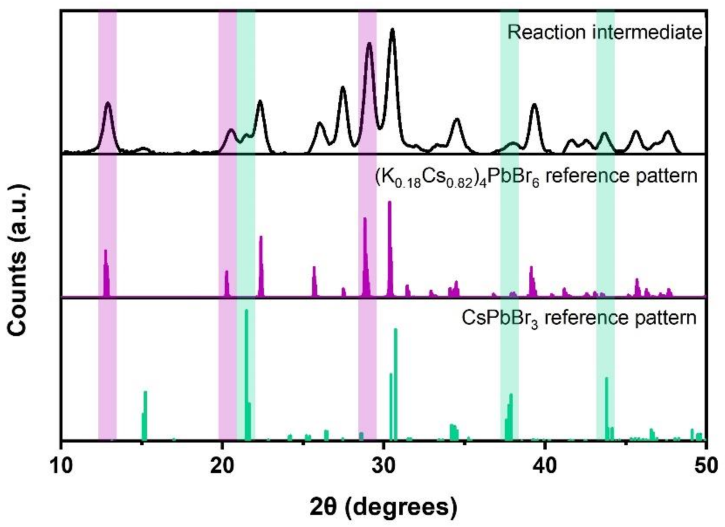

Figure S18. XRD diffraction pattern of the reaction intermediate during 0D phase $(K_{0.18}Cs_{0.82})_4PbBr_6$ nanocrystals transformation to 3D phase $CsPbBr_3$ QDs (black). The comparison with the corresponding reference patterns of $(K_{0.18}Cs_{0.82})_4PbBr_6$ (magenta) and $CsPbBr_3$ (green) indicates the coexistence of both phases during the reaction.

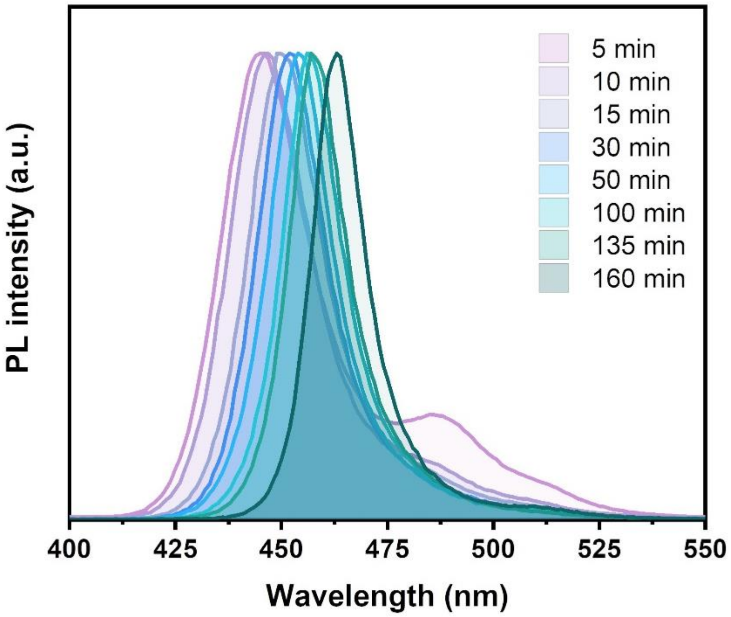

Figure S19. Normalized PL spectra of spectra of $CsPbBr_3$ QDs of different sizes obtained at different reaction times.

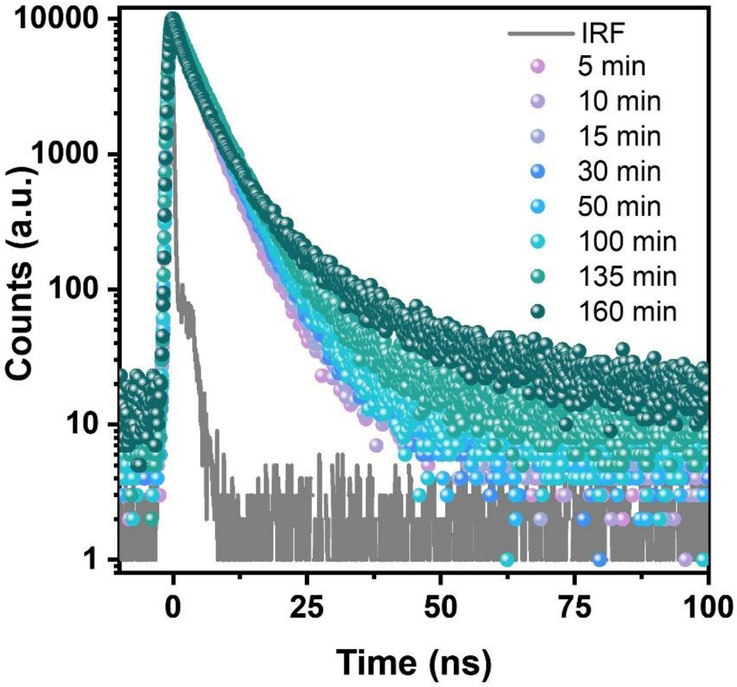

Figure S20. PL lifetime decays of $CsPbBr_3$ QDs synthesized at different reaction times obtained with a bi-exponential model.

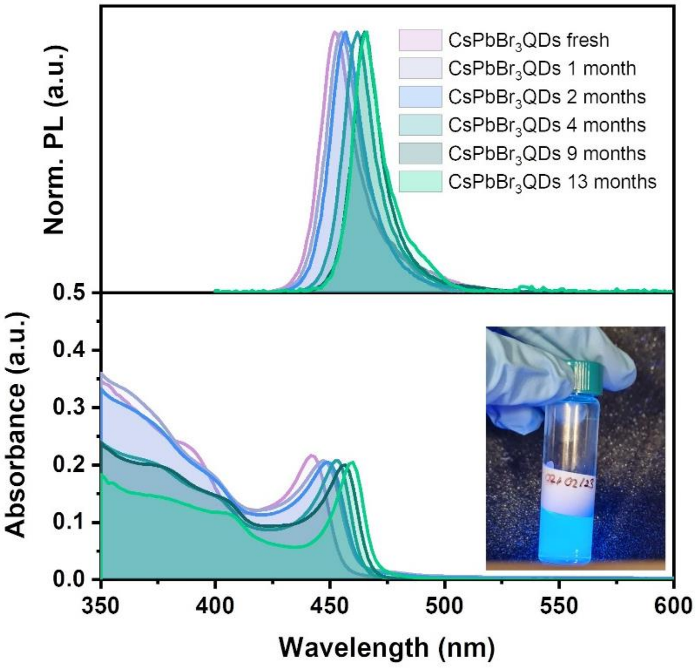

Figure S21. Absorption (bottom) and PL spectra of a colloidal solution of $CsPbBr_3$ QDs in toluene along 13 months. In the inset, photograph of the colloidal solution after 13 months.

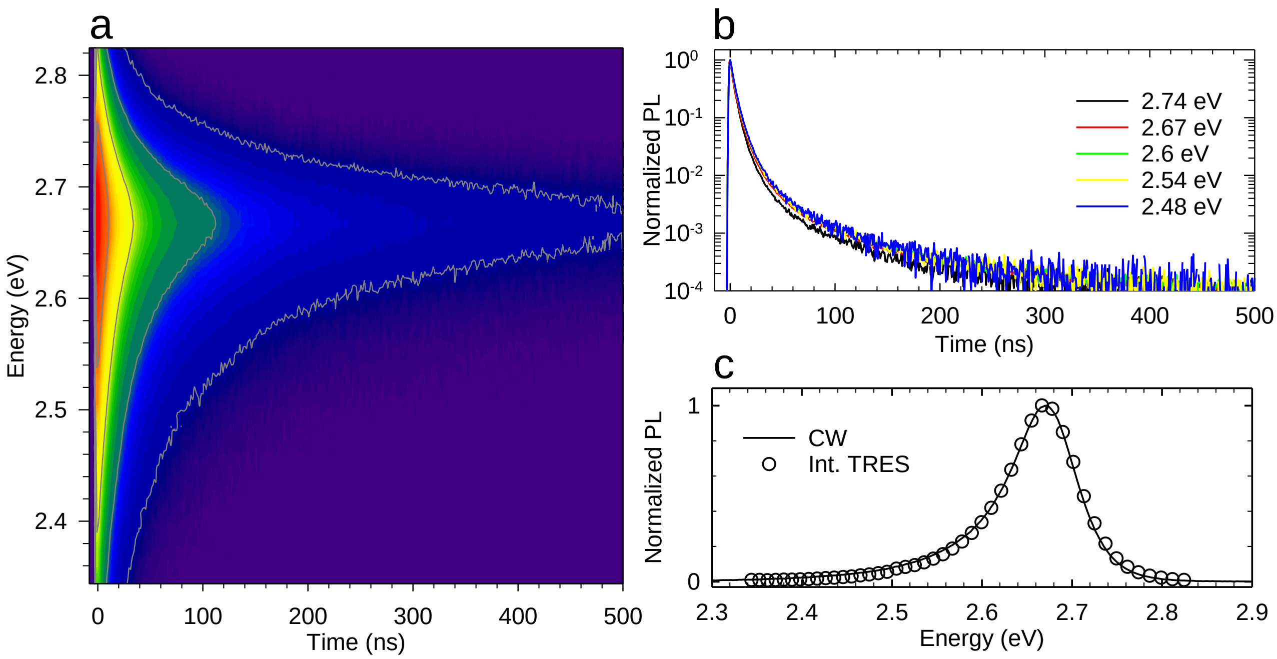

Figure S22. Contour plot of the spectrally resolved PL from $CsPbBr_3$ QDs (3.5 nm in size) under 3.05 eV excitation showing a uniform decay across the spectrum. The PL decay traces extracted at selected energies are shown in ‘b’. c) Comparison between the time integrated PL spectrum and the cw PL spectrum showing perfect overlap.

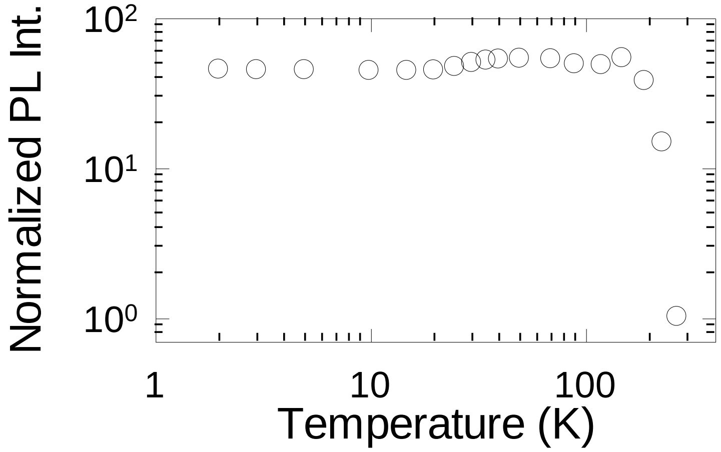

Figure S23. Temperature dependence of PL intensity for 3.5 nm $CsPbBr_3$ QDs. The data have been normalized for the value at room temperature.

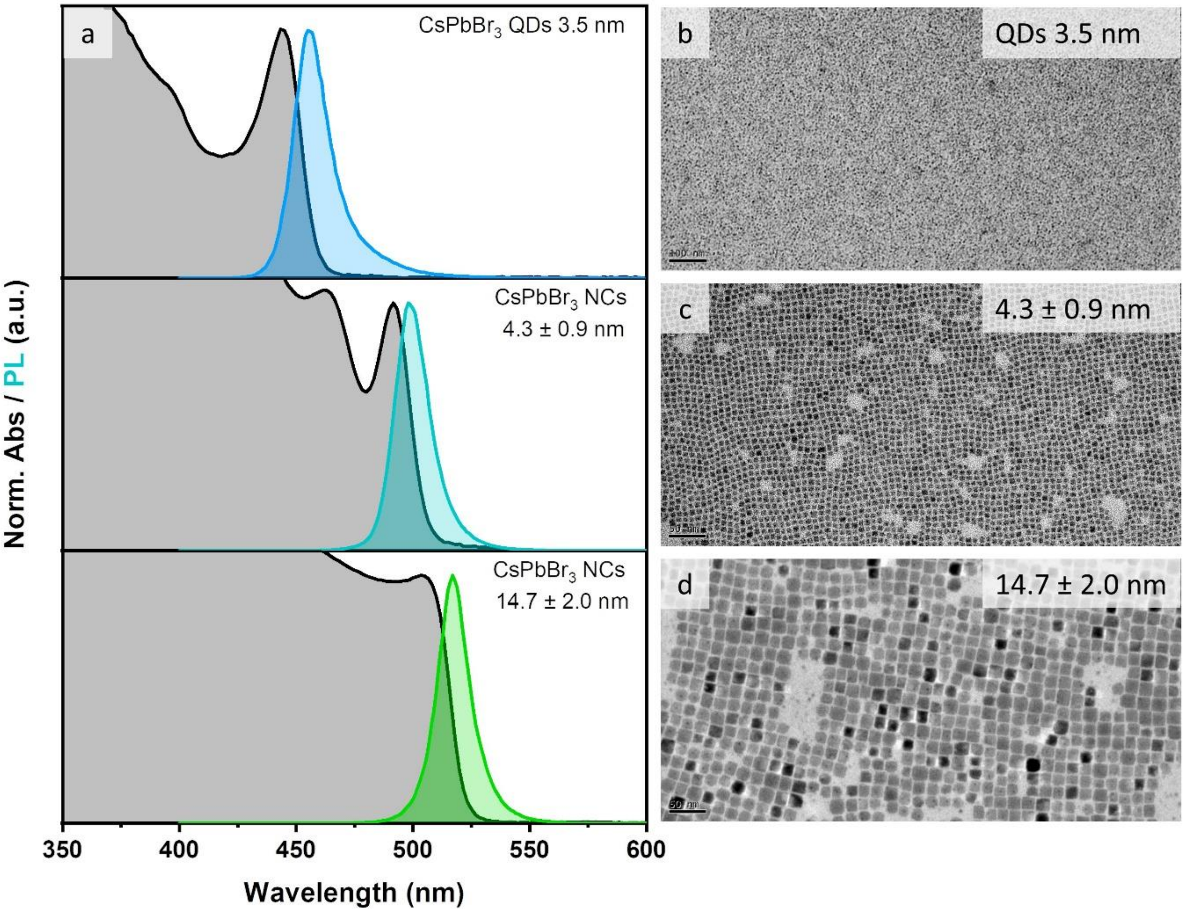

Figure S24. (a) Absorption and PL spectra of $CsPbBr_3$ QDs and nanocrystals with different lateral size. (b-d) Corresponding TEM images of 3.5 nm $CsPbBr_3$ QDs synthesized by $(K_{0.18}Cs_{0.82})_4PbBr_6$ recrystallization (b) and 4.3 nm $CsPbBr_3$ QDs (c) and 14.7 nm $CsPbBr_3$ nanocrystals (d), synthesized by a standard protocol previously reported.[2]

References

(1) Akkerman, Q. A.; Park, S.; Radicchi, E.; Nunzi, F.; Mosconi, E.; De Angelis, F.; Brescia, R.; Rastogi, P.; Prato, M.; Manna, L. Nearly Monodisperse Insulator Cs_4PbX_6 (X = Cl, Br, I) Nanocrystals, Their Mixed Halide Compositions, and Their Transformation into $CsPbX_3$ Nanocrystals. *Nano Lett.* **2017**, *17* (3), 1924-1930.

(2) Imran, M.; Ijaz, P.; Baranov, D.; Goldoni, L.; Petralanda, U.; Akkerman, Q.; Abdelhady, A. L.; Prato, M.; Bianchini, P.; Infante, I.; et al. Shape-Pure, Nearly Monodispersed $CsPbBr_3$ Nanocubes Prepared Using Secondary Aliphatic Amines. *Nano Lett.* **2018**, *18* (12), 7822-7831.

(3) Wu, P. S. C.; Otting, G. Rapid Pulse Length Determination in High-Resolution NMR. *J. Magn. Res.* **2005**, *176* (1), 115-119.

(4) Wider, G.; Dreier, L. Measuring Protein Concentrations by NMR Spectroscopy. *J. Am. Chem. Soc.* **2006**, *128* (8), 2571-2576.

(5) Grau-Crespo, R.; Hamad, S.; Catlow, C. R. A.; de Leeuw, N. H. Symmetry-Adapted Configurational Modelling of Fractional Site Occupancy in Solids. *j. Phys. Condens. Matter* **2007**, *19* (25), 256201.

(6) Hohenberg, P.; Kohn, W. Inhomogeneous Electron Gas. *Phys. Rev.* **1964**, *136* (3B), B864-B871.

(7) Kohn, W.; Sham, L. J. Self-Consistent Equations Including Exchange and Correlation Effects. *Phys. Rev.* **1965**, *140* (4A), A1133-A1138.

(8) Kresse, G.; Furthmüller, J. Efficient Iterative Schemes for Ab Initio Total-Energy Calculations Using a Plane-Wave Basis Set. *Phys. Rev. B* **1996**, *54* (16), 11169-11186.

(9) Blöchl, P. E. Projector Augmented-Wave Method. *Phys. Rev. B* **1994**, *50* (24), 17953-17979.

(10) Perdew, J. P.; Burke, K.; Ernzerhof, M. J. P. r. l. Generalized Gradient Approximation Made Simple. *Phys. Rev. Lett.* **1996**, *77* (18), 3865.
